# The Use of Mobile Assessments for Monitoring Mental Health in Youth: Umbrella Review

**DOI:** 10.2196/45540

**Published:** 2023-09-19

**Authors:** Laura Marciano, Emanuela Vocaj, Mesfin A Bekalu, Antonino La Tona, Giulia Rocchi, Kasisomayajula Viswanath

**Affiliations:** 1 Lee Kum Sheung Center for Health and Happiness, Department of Social and Behavioral Sciences, Harvard T.H. Chan School of Public Health Boston, MA United States; 2 Dana Farber Cancer Institute Boston, MA United States; 3 Lombard School of Cognitive-Neuropsychological Psychotherapy Pavia Italy; 4 Dipartimento di Scienze Umane e Sociali Università degli Studi di Bergamo Bergamo Italy; 5 Department of Dynamic, Clinical Psychology and Health Studies, Sapienza University Rome Italy

**Keywords:** mobile assessment, ecological momentary assessments, EMAs, digital phenotyping, umbrella review, youth, mental health, mobile phone

## Abstract

**Background:**

Improving mental health in youth is a major concern. Future approaches to monitor and intervene in youth mental health problems should rely on mobile tools that allow for the daily monitoring of mental health both actively (eg, using ecological momentary assessments [EMAs]) and passively (eg, digital phenotyping) by capturing individuals’ data.

**Objective:**

This umbrella review aims to (1) report the main characteristics of existing reviews on mental health and young people, including mobile approaches to mental health; (2) describe EMAs and trace data and the mental health conditions investigated; (3) report the main results; and (4) outline promises, limitations, and directions for future research.

**Methods:**

A systematic literature search was carried out in 9 scientific databases (Communication & Mass Media Complete, Psychology and Behavioral Sciences Collection, PsycINFO, CINAHL, ERIC, MEDLINE, the ProQuest Sociology Database, Web of Science, and PubMed) on January 30, 2022, coupled with a hand search and updated in July 2022. We included (systematic) reviews of EMAs and trace data in the context of mental health, with a specific focus on young populations, including children, adolescents, and young adults. The quality of the included reviews was evaluated using the AMSTAR (Assessment of Multiple Systematic Reviews) checklist.

**Results:**

After the screening process, 30 reviews (published between 2016 and 2022) were included in this umbrella review, of which 21 (70%) were systematic reviews and 9 (30%) were narrative reviews. The included systematic reviews focused on symptoms of depression (5/21, 24%); bipolar disorders, schizophrenia, or psychosis (6/21, 29%); general ill-being (5/21, 24%); cognitive abilities (2/21, 9.5%); well-being (1/21, 5%); personality (1/21, 5%); and suicidal thoughts (1/21, 5%). Of the 21 systematic reviews, 15 (71%) summarized studies that used mobile apps for tracing, 2 (10%) summarized studies that used them for intervention, and 4 (19%) summarized studies that used them for both intervention and tracing. Mobile tools used in the systematic reviews were smartphones only (8/21, 38%), smartphones and wearable devices (6/21, 29%), and smartphones with other tools (7/21, 33%). In total, 29% (6/21) of the systematic reviews focused on EMAs, including ecological momentary interventions; 33% (7/21) focused on trace data; and 38% (8/21) focused on both. Narrative reviews mainly focused on the discussion of issues related to digital phenotyping, existing theoretical frameworks used, new opportunities, and practical examples.

**Conclusions:**

EMAs and trace data in the context of mental health assessments and interventions are promising tools. Opportunities (eg, using mobile approaches in low- and middle-income countries, integration of multimodal data, and improving self-efficacy and self-awareness on mental health) and limitations (eg, absence of theoretical frameworks, difficulty in assessing the reliability and effectiveness of such approaches, and need to appropriately assess the quality of the studies) were further discussed.

**Trial Registration:**

PROSPERO CRD42022347717; https://www.crd.york.ac.uk/prospero/display_record.php?RecordID=347717

## Introduction

### Background

According to the World Health Organization (WHO) [1], 1 in 8 people worldwide lives with mental disorders, of whom >80% reside in low- or middle-income countries (LMICs) [2]. Prevalence rates are even higher in children and adolescents, with 1 in 5 young people experiencing mental health problems, which increases to 1 in 2 in the United States [3]. Overall, the economic consequences of mental health conditions are massive, with drops in productivity and indirect costs to society often higher than health care costs [1]. However, to date, only 25% of individuals in the United States with any mental illness receive psychological help [4], whereas in LMICs, this number drops to 10% to 11% [2]. Improving mental health care was already a target of the Sustainable Development Goals [5], and ongoing changes to provide adequate support for young people’s mental health should follow the *paths of transformation* outlined by the WHO in July 2022 [1]. These paths include (1) strengthening value and commitment (eg, by giving mental and physical health equal importance), (2) reshaping the environment for mental health treatment (eg, including homes, schools, workplaces, health care services, communities, and natural environments), and (3) strengthening mental health care (eg, by making mental health care affordable and accessible for all and promoting a person-centered approach) [1]. The need to reinvent mental health care is also in line with the report by the National Institute of Mental Health [3]. In particular, the report recommends that interventions to promote, monitor, and intervene require services and support that extend *beyond* clinical treatment.

In this study, we aim to revise all the available (systematic) reviews on the use of ecological momentary assessments (EMAs) and trace data in the context of mental health—and related constructs such as personality and cognition—in youth, broadly defined according to the included reviews as children, adolescents, and young adults. In the following sections, we aim to introduce the concepts of mobile assessments in mental health care and provide more detailed definitions of digital phenotyping and EMAs.

### Mobile Assessments in Mental Health Care

The newly introduced policy by the WHO recommends an increase in self-management or self-care tools by, for example, using mobile devices. Mobile health (mHealth) apps help diminish the stigma [6,7] and discrimination related to mental health issues and overcome the problem of inaccessible or unaffordable therapies, especially among minority groups and immigrants [7,8]. Hence, mHealth interventions are a promising avenue to bridge the gap between seeking help and accessing mental health resources [9], particularly in young people [10-12], who are experiencing the highest prevalence of psychological problems but who are also the heaviest smartphone users [13]. Health care productivity can be increased using self-care [14], which has proven to be cost saving and has cost-effective benefits [15]. In addition, mobile tools allow for the collaborative use of data, thus improving clinical decision-making and fostering a tailored approach to health [16,17]. At the same time, patients are increasingly interested in using their smartphones and mental health care apps [18]. The research area of *precision medicine* [4-6] stresses the use of digital and health information to monitor well-being and prevent and treat illness at a highly personalized level based on a *prediction*, *prevention*, *personalization*, and *participation* approach [19-21]. Considering that 75% of lifetime mental disorders emerge by the age of 25 years, it is critical to promote prevention in childhood and adolescence. Defining underlying mechanisms, together with risk and resilience factors, will likely result in earlier prevention and targeted intervention [16]. To note, the precision medicine market targeting health is expected to reach US $738.8 billion by 2030 [22]. That said, the future of mental health care seems to rely on digital approaches that allow for the monitoring of people’s health daily both actively and passively by capturing individuals’ data continuously without additional effort. This approach is expected to become increasingly relevant, as pointed out by the “extended” version of phenotyping [23], that is, the *digital phenotyping* approach, described as the “moment-by-moment quantification of the individual-level human phenotype in situ using data from personal digital devices, in particular smartphones” [24]. As stated by Montag et al [25], “the area of *digital phenotyping* could be seen as being part of a larger scientific movement labeled *Psychoinformatics*, where computer science and psychology collaborate to understand the human mind. This interdisciplinary research project is still young, but rapidly evolving.”

### Digital Phenotyping and EMAs

Mobile assessments and interventions offer promising opportunities to monitor and assess mental health symptoms in 2 ways. First, smartphones and wearable technology allow for the gathering of data passively and uninterruptedly (ie, trace data [26]) owing to their unobtrusiveness combined with their pervasiveness. Sensors embedded in smartphones, such as digital cameras, microphones, GPS, accelerometers, gyroscopes, Wi-Fi, Bluetooth, and light and sound sensors, automatically collect users’ data, including physical states and behaviors (such as motor activity, heart rate, temperature, and other physiological variables) and behavioral data that capture physical location, patterns of smartphone use, and speech patterns. Trace data allow for the study of the intersubject variability and explore heterogeneity within and between users in a way that was not possible before. In addition, real-time data collection in a natural environment allows for the gathering of information about the context and events, thus augmenting our understanding of the dynamic nature of mental health on a continuum from ill-being to well-being.

The second way to assess and monitor mental health is through the active participation of the user. Owing to specific mobile apps, it is possible to actively monitor subjective experiences using EMAs, which require the user to answer brief questionnaires during the day. EMAs offer advantages over traditional survey methods [18], such as overcoming retrospective recall biases (as questions usually refer to the current state), augmenting ecological validity [27], the possibility of modeling within-person changes in self-reported information, the assessment of temporal dynamics among related constructs, the capacity to monitor treatment or progress in real time [28], and the valuation of phenomena as they are happening [29]. EMAs allow for the questioning of participants while they are in a natural environment, thus tackling microprocesses influencing behavior [29]. The opportunity to assess fluctuations and changes in affective dynamics over time as dynamic processes represents one of the greatest strengths of EMAs [30]. For example, recall of mood states differs depending on other factors such as cognitive styles, and people with mood disorders tend to recall negative experiences and emotions, thus exaggerating the presence of symptoms [31].

In addition to passive and active data, digital phenotyping also involves using metadata [32] (ie, hybrid data), including information on the user’s interaction with the app (eg, time taken to complete a survey). To summarize, owing to the richness of this approach, EMAs and trace data can add significant information to conventional methods by providing the individual information necessary to make personalized predictions and interventions.

### Rationale

Despite the increasing interest and constant development of mobile systems to assess and monitor mental health, the literature is still heterogeneous, and there is a lack of a comprehensive framework. Existing mapping reviews convey information on mobile apps used to monitor mental health [33-35], with a focus on user-adjusted analyses (eg, examining active users) [36] and sensing and computing technologies for digital phenotyping of mental health [37]. However, none have tried to summarize the advantages and pitfalls of mobile assessments and interventions conveyed through EMAs and trace data by summarizing the increasing number of existing reviews on the topic.

With the increasing number of mental health apps and the use of mobile data, coupled with prevalence data showing a mental health crisis in young people [38], it is now crucial to inform future research, practice, and policy making and regulations. Conducting an umbrella review of the literature will allow us to explicitly and systematically summarize results and methods, compare previous reviews’ findings, and guide future research on this emerging field [39].

Hence, this umbrella review systematically describes all reviews investigating the use of EMAs and trace data in the context of mental health in children, adolescents, and young adults. With this high-level synthesis, we aimed to report (1) the main characteristics of the existing reviews (in terms of methodology and participants) and (2) collected trace and EMA data and the mental health domains investigated; (3) describe the main results; and, finally, (4) outline promises, limitations, and future directions.

## Methods

### Overview

The systematic literature review was conducted according to the PRISMA (Preferred Reporting Items for Systematic Reviews and Meta-Analyses) guidelines and rules for conducting umbrella reviews [39-41]. A PRISMA checklist is presented in Multimedia Appendix 1 [8]. The protocol is registered in PROSPERO (CRD42022347717). In the following sections, we highlight the search strategy, inclusion and exclusion criteria, data extraction and preparation procedure, and methodological quality assessment.

### Search Strategy

On January 30, 2022, a systematic literature search was carried out in 9 scientific databases: Communication & Mass Media Complete, Psychology and Behavioral Sciences Collection, PsycINFO, CINAHL, ERIC, MEDLINE, the ProQuest Sociology Database, Web of Science, and PubMed. Keywords included terms such as “EMA* OR digital phenotyp*” AND “review*” AND “young*.” The complete list of search terms used to find eligible articles is presented in Multimedia Appendix 2 [11,42-61]. All articles published after 2007 (when the first iPhone was released) were searched for. An additional hand search was carried out in July 2022 by screening the first 100 entries in Google Scholar combining the same keywords. We imported all entries into Zotero (Corporation for Digital Scholarship) to automatically exclude duplicates. The first author carried out the initial title and abstract screening. In total, 2 authors (EV and ALT) carried out the full-text screening independently in Excel (Microsoft Corp). Discrepancies were resolved through a consensus meeting.

### Inclusion and Exclusion Criteria

After title and abstract screening, a study was retained if it met the following predefined eligibility criteria: (1) using a (systematic) review approach to (2) summarize the literature on EMAs and trace data in the context of mental health—including related psychological constructs such as personality or cognition as they are both associated with mental health [62,63]—(3) including studies that used mobile tools, and (4) with a specific focus on young population groups (ie, children, adolescents, and young adults). We included only reviews in English and published in peer-reviewed journals. Reviews were excluded if they were focused on the use of digital gaming or virtual reality in the health context; if they were focused on physical activity, sleep, or eating behaviors without a reference to mental health; if digital interventions were carried out as part of another setting, such as cognitive behavioral therapy or web-based information or in the context of psychoeducation or psychological websites and no reference was made to EMAs or digital phenotyping; and if they were primarily focused on populations with health problems, such as cancer or diabetes, or older adults. We also excluded conference papers and book chapters.

### Data Extraction and Preparation

For each study that met the inclusion criteria, we collected information on the following: title and journal where it was published, year of publication, review aim, included databases, date of research, number of included studies, whether the review was systematic or not, number of participants per included study, number of participants in total included in the review, recruitment of participants, mean age, other information about the included population, type of population (children, adolescents, or young adults), countries in which the included studies were conducted, whether the review was focused on tracing or intervention studies, duration of the included studies, the intensity of data collection per day, the mobile tool used (eg, smartphones, sensors, or others), details on other included tools, information on the smartphone apps used, the presence of EMA or trace data or both, more details on the EMA and trace data collected, description of data analysis, presence of control groups in the included studies, presence of data validation (eg, presence of other data to validate the information collected), the psychological symptoms and behaviors that the studies wanted to monitor or intervene on, the general mental health category of the included conditions, the presence of compliance rates with EMAs, the presence of risk-of-bias assessments of the included studies, and the main results obtained. In total, 2 authors collected the included information, and 2 other authors checked the accuracy of the extrapolated data.

### Methodological Quality Assessment

To assess the methodological quality of the systematic reviews, we used the AMSTAR (Assessment of Multiple Systematic Reviews) tool developed by Shea et al [64]. The AMSTAR checklist includes 11 criteria to assess the quality of a systematic review: (1) the presence of an “a priori” design provided (ie, research question and inclusion criteria), (2) the presence of 2 coders for study selection, (3) the presence of a comprehensive literature search performed through at least 2 electronic sources, (4) whether the status of publication (ie, gray literature) was used as an inclusion or exclusion criterion, (5) whether a list of included or excluded studies was provided, (6) whether the characteristics of the included studies were provided in aggregate forms such as tables and figures, (7) whether the scientific quality of the included studies was assessed and documented, (8) whether the scientific quality of the included studies was used appropriately in formulating conclusions, (9) whether the methods used (eg, meta-analysis) to combine the findings of the studies were appropriate, (10) whether publication bias was assessed, and (11) whether any conflicts of interest were stated. Each systematic review was assessed using this checklist; answer options were *Yes*, *No*, *Can’t answer*, and *Not applicable*. One author carried out the risk-of-bias assessments (EV), and a second author (GR) checked the information independently. Doubts and conflicts were resolved through a consensus meeting with a third author.

## Results

### Overview

**Figure 1:**
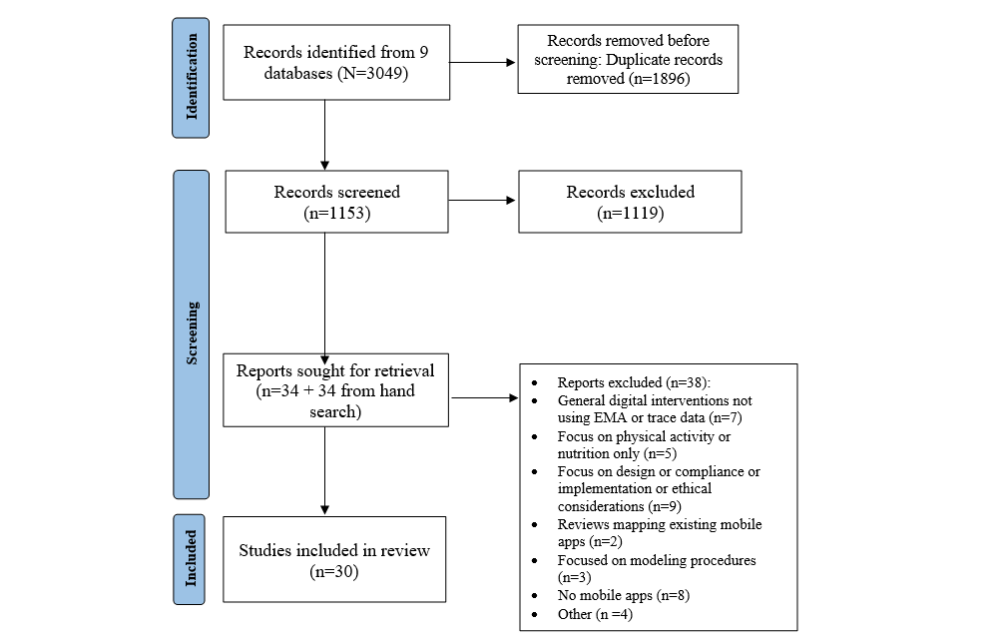
PRISMA (Preferred Reporting Items for Systematic Reviews and Meta-Analyses) flowchart. EMA: ecological momentary assessment.

The initial search returned 3049 entries, which resulted in 1153 (37.82%) after duplicate removal. After title and abstract screening, 97.05% (1119/1153) of the entries were removed as they were off-topic. A total of 34 studies were retrieved for full-text screening along with an additional 34 studies from the hand search. At this stage, of the 68 reviews, 7 (10%) were excluded as they focused on general digital interventions (eg, web-based therapies and psychoeducation) without using trace or EMA data; 9 (13%) tackled issues of design, compliance, implementation, or ethical considerations; 8 (12%) did not include a mobile assessment; 5 (7%) focused on outcomes such as physical activity, sleep, or nutrition; 2 (3%) were mapping reviews describing existing mobile apps for mental health; 3 (4%) focused on statistical modeling; and 4 (6%) were excluded for other reasons, such as EMAs carried out by caregivers or inclusion of paper-and-pencil diaries only. A total of 30 reviews were finally included in this umbrella review, of which 21 (70%) were systematic reviews and 9 (30%) were narrative reviews. A comprehensive description of the screening process is presented in the PRISMA flowchart (Figure 1). In the following sections, we provide a description of the included reviews by providing information on participants; mobile tools used; trace and EMA data included; mental health conditions investigated; main results; and more specific information on the data analysis procedure, cross-validation of data, the reviews’ quality, and participant adherence. Finally, we descriptively summarize the included narrative reviews.

### Description of the Included Reviews

#### Overview

The included reviews were published between 2016 and 2022. We will here systematically summarize the results of the systematic reviews (n=21). Of the 21 systematic reviews, 12 (57%) reported information on the countries of the included studies—they were mainly conducted in North America, Europe, and Asia, whereas middle-income countries such as Mexico [42] and Brazil [43,44] were less represented. A total of 71% (15/21) of the reviews summarized studies that used mobile apps for tracing, 9.5% (2/21) summarized studies that used mobile apps for intervention, and 19% (4/21) summarized studies that used mobile apps for both intervention and tracing. The number of studies included in the systematic reviews ranged from 12 [45] to 118 [46], with a median of 35.

A summary of the characteristics of all the included reviews is reported in Tables S1-S3 in Multimedia Appendix 3 [11,42-61].

#### Participant Characteristics

The average number of participants in the included studies in the reviews ranged from 23 [42] to 164 [47]. However, the sample sizes of the included studies varied greatly, for example, ranging from 16 to 2167 [48], 62 to 84,451 [49], 10 to 8242 [50], and 15 to 31,302 [51]. In general, most of the reviews included young adults (11/21, 52%), adolescents and young adults (6/21, 29%), and children and adolescents (4/21, 19%); however, the mean age of the included participants was reported only in 29% (6/21) of the reviews. Overall, 29% (6/21) of the reviews reported details on how participants were recruited to join the studies. In particular, the reviews described that the included studies recruited participants via web using convenience and snowball sampling [42,51-53]. Participants were also recruited from a target population [51], especially in clinical settings [54,55]. Some reviews (5/21, 24%) included only studies with participants meeting the clinical criteria for mood disorders or depression [43,44,47,52,56] or studies focusing on psychosis and schizophrenia [42,46,53,54,57,58]. In contrast, in other cases the focus was on healthy participants [45,51] or specific populations such as college students [11], and some (3/21, 14%) used mixed populations from clinical and nonclinical settings [44,46,59]. Usually, other information on the diversity of samples in the included studies in the reviews (eg, ethnicity and socioeconomic status) was lacking.

#### Mobile Tools Used

The mobile tools included in the reviews were smartphones only (8/21, 387%), smartphones and wearable devices (6/21, 29%), and smartphones with other tools (7/21, 33%). In particular, wearable devices included actigraphy-based wrist-worn devices such as Fitbit and Microsoft bands [43], biosensors, PDAs, palm-top computers, handheld computers (with an additional accelerometer to wear on the arm, chest, or hip or a heart rate meter), smartwatches [11,45,48,51,56,60], heart patches, and digital rings [44]. In addition, in some cases, wearables were used for assessment, monitoring, or delivery of an intervention (or, at least in part, combined with clinical sessions or ongoing input from a clinic) [54]. Other information combined with EMA and trace data was social media data such as Facebook data [49] but also neuroimaging data, including magnetic resonance imaging (MRI), functional MRI, electroencephalogram, and positron emission tomography [57] and cortisol levels through saliva [60]. One review included studies that combined different sources, such as actigraphy, functional MRI, salivary samples, pupil dilatation, and pupillary responses [47]. Finally, one review focused on ecological momentary interventions (EMIs) and included studies in which EMIs were coupled with psychological therapies, including mobile cognitive behavioral therapy, mobile interpersonal psychotherapy, virtual reality, and biofeedback [59].

A total of 66% (14/21) of the reviews reported on the duration of the studies, which, among all the reviews, ranged from 1 [45] to 17 weeks [53] on average. However, in 2 cases the included studies’ duration ranged from 1 day to 1 year [43,54]. In addition, in the case of EMAs, the intensity of data collection across all the included reviews ranged from 2 to 12 times per day [51,55,60]. When looking at specific included studies, some collected data more often, with an average of 10 times daily [54], or less frequently, approximately 3 or 4 times daily [11,45], down to 2.8 in the case of EMIs [59]. In one case, more specific information on the intensity of data collection was mentioned [57].

More details on the app used were reported in 57% (12/21) of the reviews. In particular, a review mentioned specific apps used to monitor mood symptoms in children and adolescents [47], such as Daybuilder, Mobile Mood Diary, and the Atria Convergence Technologies Ltd app. However, most available mental health apps were not supported by evidence-based research and, thus, did not follow evidence-based guidelines for treatment. Benoit et al [53] reported that 25 unique apps in 40 studies were used to collect data. Camacho et al [57] reported a list of app names. de Vries et al [51] highlighted that only 5 (4.2%) apps out of 118 included studies were used in multiple publications to answer different research questions. In 1 of the reviews [42], 89% of the studies used Android operating system apps, and another 1 [46] reported that 56.7% of the studies developed apps for Android smartphones only and just a few developed apps for both Android and iOS. In addition, De Angel et al [43] reported different wearables used, such as Actiwatch 4 and Empatica. In addition, wearable tools to characterize depression in children and adolescents were reported by Sequeira et al [56] and included apps such as Acer Liquid Z200, Mobiletype, CopeSmart, PETE, SOLVD, eMate, iYouVU, Daybuilder, and StudentLife. In the reviews that included intervention studies, a long list of apps used was included [50], and the apps usually exhibited more than one function. In particular, apps designated not only to collect data but also for mobile interventions targeted client communication, client-to-client communication, and information services. A study [49] used myPersonality data, a Facebook application for psychometric tests. Interestingly, in the review by Moore et al [45], each study reported developing apps for cognitive assessments in-house, and none of the tests were available in app stores.

### Trace and EMA Data in the Included Reviews

A total of 29% (6/21) of the included systematic reviews focused on EMAs (including EMIs), 33% (7/21) focused on trace data, and 38% (8/21) focused on both.

#### EMAs and EMIs

In particular, reviews on EMAs summarized the use of this methodology to monitor mood symptoms and also to evaluate the usability, feasibility, and acceptability of this technology among young people with mental health problems. For example, EMAs were used to investigate daily emotional dynamics of child and adolescent depression, such as negative affect variability; sociability, including patterns of interactions with peers and parents; and events of corumination, but also how the use of substances such as caffeine or social media consumption influence young people’s mood [47]. At the same time, EMAs were used to evaluate treatment responses in adolescents with mood (and anxiety) disorders. Thunnissen et al [60] also focused on young people’s psychopathology and its correlation with daily life and parent-child interactions. Hence, the included studies used EMAs to assess attention-deficit/hyperactivity disorder, autism spectrum disorder, mood disorders, anxiety disorders, and eating disorders. Similarly, another review [52] collected information on app-based EMAs, which were used to monitor mood states and daily emotion dynamics, as both measurement and intervention tools, with the aim of increasing affective awareness and reducing depressive symptoms as well as identifying risk factors and exploring EMA use outside the therapy context.

Kivelä et al [55] studied how EMAs have been used to investigate suicidal thoughts and behaviors in adolescents and young adults by asking questions about the frequency of suicidal ideation and its duration, both passive and active thoughts, and reported on their correlation with depression scores. One review [45] used EMAs to convey cognitive assessments in clinical and nonclinical populations and observed that, usually, a single mobile cognitive task was given for the duration of the study, with only a few included studies administering more than one task. The tasks were unique, and no 2 studies used the same version. Along the same lines, Versluis et al [59] focused on studies using EMIs for ill-being symptoms (anxiety, depression, and perceived stress) and positive psychological outcomes. They investigated a range of different EMA-based interventions, of which one-third were in combination with cognitive behavioral therapy, acceptance and commitment therapy, mindfulness, behavioral activation, relaxation, interpersonal therapy, dialectical behavior therapy, cognitive bias modification, and self-management or monitoring strategies. Most included studies investigated whether EMI could be effective without face-to-face therapy.

#### Trace Data

Some reviews focused only on trace data. For example, information on phone sensors and analytics was used by Benoit et al [53] to assess symptoms of clinical people at high risk of schizophrenia, psychosis, and bipolar disorder or its early course and long-lasting symptoms. In other cases, trace data were used to explore the correlates of health and well-being in general [42,46]. In addition, wearables were used in the context of depression [43]—in this specific case, more emphasis was placed on sleep patterns, and trace data were collected on sleep quality and efficiency and for measuring circadian rhythms. With the aim of evaluating mental health mobile apps for transition-age youth, Lo et al [50] collected different metrics such as the number of clicks, number of features used, number of log-ins, number of modules completed, and session duration, whereas Marengo and Montag [49] reviewed how Facebook data have been used to predict Big 5 personality traits by collecting user demographics and digital footprints in terms of activity statistics (eg, number of posts; number of friends; and number of received likes, comments, and user tags), liking activities (eg, likes expressed to specific Facebook pages), characteristics of language in text (eg, features extracted using closed- and open-vocabulary approaches), and features from pictures (eg, features extracted from uploaded pictures). To explore associations between sensor data and psychiatric disorder symptoms and support the mobile therapeutic attention for patients with treatment-resistant schizophrenia approach [65], the following features from sensor data were used [58]: app number and duration of incoming, outgoing, and missed calls; number of incoming and outgoing SMS text messages; time spent at home and in other places; and time spent sleeping measured using physiological heart rate.

#### Trace and EMA Data

The review by de Vries et al [51] included trace and EMA data to study well-being in the general population. In this case, 58.5% of the studies used trace with EMA data. In addition, the included studies investigated well-being through EMAs in diverse contexts (eg, in relation to physical activity and sedentary behavior, alcohol and food consumption, sleep, mind wandering, cognitive processes, and well-being fluctuations). In this review, well-being (and happiness) was mainly measured daily using 1 item. Similarly, Melcher et al [11] summarized current practices in digital phenotyping to monitor mental health in college students and found that 52% of the studies used active data collection in addition to passively collected data. In both cases, trace data included an accelerometer, GPS location, call logs, internet use, browser history, mobile phone use (including app use), heart rate variability, light sensors, microphones, events, screen use, and Wi-Fi. Similarly, Kulkarni et al [61] summed up the literature on the potential of smartphone sensing in health care to provide an overview of the existing practice. In this review, 84.5% of the included studies used both actively and passively sensed data. Trace data mainly collect information from the accelerometer and GPS, and some looked at app use but also different smartphone sensors to map contextual information, such as keyboards to analyze the typed text, barometers for the type of activity, and internet and social media data (such as Twitter and Instagram) to obtain information on mental health. EMA included daily assessments of factors such as food intake and mental health, such as mood, stress, and loneliness; however, questionnaires were usually not standardized across studies. Zarate et al [44] reported the use of digital footprints to examine, diagnose, and monitor depression using the aforementioned trace data and, in addition, more advanced smartphone keystroke metadata, speech technology, and facial recognition. In this case, trace data were combined with self-reported changes in affect, physical health, and cognition and risk and protective factors of the intervention outcomes. A more specific focus on children and adolescent mood disorders was taken by Sequeira et al [56], who collected trace data, including passive analytics but also information through wearables, to measure individuals’ activities and locations, whereas in the same review, studies used EMAs for both physical (eg, energy, alcohol use, cannabis use, quality and quantity of sleep, diet, and quantity and type of exercise) and mental (eg, affect, mood, stress, life events, help seeking, depression, anxiety, and health coping strategies) outcomes.

Camacho et al [57] summarized the evidence on the interconnection of neuroimaging and smartphone data. In doing so, they relied on the Research Domain Criteria (RDoC) domains in the context of translational neuroscience to understand how digital measures can bridge information from neuroscience with behaviors and self-reported data. In particular, the included studies focused on the assessment of negative and positive valence systems, social processes, arousal and regulatory systems, sensorimotor skills, and cognitive systems. Cognition through intensive data collection was explored in the review by Weizenbaum et al [48], in which intraindividual variability in cognition was linked to internal states (eg, affect motivation and alertness) and external contextual variables (eg, time of day, social environment, physical surroundings, and physical activity) using game-like mobile assessments coupled with sensing data. In particular, individuals’ cognition was measured using tasks tackling different cognitive processes such as semantic reasoning, memory, executive function, language, motor and processing speed, and reaction time.

Jameel et al [54] summarized studies collecting trace and EMA data in the context of interventions for people with several mental illnesses. Hence, trace data were used for both assessment and monitoring through mobile phones or wearable smartwatches. The collected information included digital social communication (via phone call and messaging analysis) and activity levels (via GPS data), whereas EMAs were mainly used to monitor or promote self-monitoring, providing feedback to enhance daily functioning and help reach daily goals. In addition, in this case, EMAs were embedded within a broader clinical intervention or in addition to clinical treatment (hence, they were called “blended”) to complement web-based psychoeducational modules or web-based therapy sessions.

### Mental Health Conditions Investigated

The included reviews focused on symptoms of depression (5/21, 24%); bipolar disorders, schizophrenia, or psychosis (6/21, 29%); general ill-being (5/21, 24%); cognitive abilities (2/21, 9.5%); well-being (1/21, 5%); personality (1/21, 5%); and suicidal thoughts (1/21, 5%).

Regarding depression, 2 studies focused on depressive symptoms in children and adolescents [47,56]. One review mainly looked at how self-monitoring through EMAs augmented self-awareness of mood symptoms [52] or general characteristics such as within-day mood changes, cognitive capacity, sleep-wake cycles, physical activity, social interactions, and depression severity [43,44]. When bipolar disorders were considered, they included type-1 and type-2 bipolar disorders. Details were collected on daily functioning, including activities and social interactions, and impairment experiences but also on the feasibility and acceptability of mobile apps and health and mHealth interventions [54], together with detecting the clinical course of illness at an early or later stage [53] and in combination with stress and anxiety conditions [46,58]. Camacho et al [57] used the National Institute of Mental Health RDoC developed by the US National Institute of Mental Health, which combines various levels of information, from genomics to behaviors, to describe mental health processes, problems, and illnesses, thus offering a general framework to understand human behavior. Reviews on general mental health problems usually assessed anxiety disorders, depressive symptoms, stress, bipolar disorder, schizophrenia, borderline personality disorder, bulimia, quality of life, chronic health, and mental health also using the same RDoC [60], whereas de Vries et al [51] summarized the literature on (subjective) well-being, happiness, quality of life, life satisfaction, and positive affect.

### Main Results of the Included Reviews

The main results of the included systematic reviews are summarized in Table 1.

**Table 1:** Summary of the results of the included systematic reviews (n=21).

Study ID	Study, year	Summary of results
1	Baltasar-Tello et al [47], 2018	Most of the studies demonstrated that EMA^a^ protocols were useful in analyzing affective symptoms in real-life environments. Participants were able to report and rate their affect at the moment—adolescents with depression were less likely to report sadness and irritability as compared with controls in real time; however, they were more likely to report these symptoms retrospectively. These findings explain why adolescents with depression may exaggerate mild negative events, amplifying recall and generalizing the negativity to other experiences. EMAs allowed for relating the type of media use to symptoms of depression. It also allowed for the prediction of pharmacological treatment response as well as the determination of relationships between affect and sleep or affect and caffeine consumption. EMA also permitted the evaluation of problem-solving, ruminative contents, social interactions, and emotions and how they vary. To improve adherence, multiple reminders are needed.
2	Beames et al [52], 2021	Affective awareness is important in the prevention and early intervention of depression, and EMAs can be used as a therapeutic tool to increase awareness and indirectly reduce depression. The indirect effect was found with subclinical and clinical samples, suggesting utility for early intervention and treatment. To have beneficial effects, EMAs should focus not just on the what but also on the why and how negative mood is elicited (eg, emotional triggers and how to deal with them). Affective awareness provides a foundation for other coping skills.
3	Benoit et al [53], 2020	Of the 51 psychiatry-related publications using digital phenotyping to analyze passively collected data, only 1 specifically focused on clinical high-risk or first-episode psychosis; the others focused on bipolar disorder. As the data and methods used in schizophrenia and bipolar disorder are highly translatable and relevant to the earliest stages of psychotic disorders, the results highlight an area of immediate opportunity for advancing research and clinical insights. Within ML^b^ studies, the smaller average of 23 participants completing each ML study showed that there is scope for using algorithms such as deep reinforcement learning that are capable of learning patterns in smaller data sets. To translate ML methods successfully, available data sets must have a sufficient number of examples from which the algorithm can learn.
4	Camacho et al [57], 2021	Most of the studies involved fMRI^c^ and used the RDoC^d^ framework focused on negative valence systems. Digital phenotyping studies can elucidate the links between dynamic snapshots of behavior and metadata, such as unlock time, which may provide more objective, time-stamped data than subjective self-reports. EEG^e^ results revealed that interacting with technology, such as through SMS text messaging, is associated with alterations in brain networks. Results also suggest that digital tools may be able to inform the relationship among brain function, peer interactions, and anxiety. Preliminary findings revealed a positive relationship between brain connectivity in the subgenual cingulate cortex (a region involved in depression) and smartphone screen time (ie, the greater the phone use, the stronger the neural connectivity). Food cues were associated with a significant increase in BOLD^f^ response on the fMRI in the left amygdala, indicating that neural reactions to food cues may play a role in the unique craving and binge eating experiences of an individual. Some behaviors correlated with ventromedial prefrontal cortex and amygdala connectivity (eg, duration of the conversation, time of sleep onset, and the number of phone unlocks). Findings indicated that there was a negative association between the tendency to make shortsighted decisions or show limited self-control in both real-world and research conditions and neural modulation signals in the ventromedial prefrontal cortex when predicting long-term consequences. However, all the studies were cross-sectional, reflecting a lack of temporal assessment of changes in brain structure.
5	Cornet and Holden [42], 2018	A number of smartphone passive sensing strategies for data collection, processing, and use were reported in all the studies, including ML or just-in-time processing and feedback. Studies concluded that smartphone passive sensing was more accurate and less intrusive compared with self-report measures. A smartphone-based passive sensing approach for health and well-being is synonymous with the concept of minimally disruptive medicine. Passive sensing can ease—or, minimally, not add to—“work that is delegated to patients and their families” by facilitating difficult tasks such as self-monitoring or daily logging. Passive monitoring is less likely to disrupt a person’s thoughts and activities than diaries and paper questionnaires. Currently, smartphones are a part of normal routines and appear less disruptive. Smartphones can also be programmed to collect social and personal data, call data, SMS text messages, and contacts. In addition, smartphones have the advantage of multifunctionalities (calling, data service, and settings control), internet connectivity, advanced processors, and high-resolution display.
6	De Angel et al [43], 2022	Most of the studies were explanatory in nature. This means that many different versions of the same feature may have been generated or the studies did not transparently describe and justify feature selection and its association with depression, thus lacking generalizability. Researchers should provide a description of the feature in the paper or supplementary materials that is sufficiently clear to allow for appropriate reproducibility. It is also likely that the included studies showed publication bias as they typically emphasized “positive” findings over “negative” ones. Preregistering would be one way of reducing this bias. Nuance is required in interpreting the presence or absence of a relationship between specific signals and depressed mood. Finally, there is also a lack of clear discussion regarding the extent to which these digital devices are the best valid and reliable tools to detect behaviors of interest. Although some behaviors may appear relatively simple to infer from single sensors, such as GPS sensors to infer location and accelerometry as a measure of movement and physical activity, there are still validity and reliability concerns surrounding them.
7	de Vries et al [51], 2020	Momentary well-being fluctuated daily and weekly, with, on average, higher well-being in the evenings and weekends. However, often both daily and weekly fluctuations disappeared when the type of place, physical activity, or other environmental variables were controlled. Being in a natural environment in daily life (eg, walking in the park) and physical activity throughout the day were associated with positive affect and higher well-being. Working was ranked lowest in happiness levels, but employees showed fluctuations based on the task, where they worked (office, home, or somewhere else), whether they were alone or with others, the time of day or night, and personal characteristics (eg, lower well-being associated with working when married but higher well-being associated with having children). Work stress is negatively related to positive affect. The effects of mind wandering on positive affect are inconclusive, with a negative and no relation. Eating is related to positive affect, with stronger effects for vegetable consumption and snacks. Drinking alcohol is related to higher momentary positive affect on average, but this increased well-being does not last or spill over to other moments. Sleep and well-being were related, with a stronger effect of sleep on mood than of mood on sleep quality. Furthermore, on average, positive affect was associated with faster visual search reaction times but not with other cognitive measures. Focusing attention on well-being by completing multiple questionnaires about well-being for a few weeks augmented well-being. Furthermore, single studies showed that exposure to fitness inspiration on the web and social media use were related to lower happiness, whereas listening to music and watching soccer were related to higher momentary happiness on average. Walking and cycling were better for mood than sitting in a bus or car. Finally, the studies predicting mood and well-being based on objective data such as phone use were successful in 55% to 76% of their mood predictions.
8	Jameel et al [54], 2021	A total of 23 studies were categorized as assessment and monitoring studies, and 15 were categorized as intervention studies. Most studies showed good levels of feasibility, acceptability, and potential to support daily functioning and recovery-related activities. Most studies were of low methodological quality. Active assessment and monitoring, if used in the early stages of illness, could help empower people with serious mental illnesses to engage in self-management activities and prevent chronicity. Passive assessment may allow the clinician and patient to gain insights from daily life with minimal effort on the participant’s behalf. Passive data collection can be used for long periods, collecting information frequently and with greater precision. This method is also less likely to change the behavior being studied, subsequently reducing the Hawthorne effect. Passive data could be used to indicate personalized warning signs of relapse, such as a shift in social behavior or daily activity. The insights garnered from mHealth^g^ devices can also be used to bridge links between clinical settings and the patient’s everyday life—mHealth technology might support the transfer of skills, strategies, and insights gained via more traditional methods of intervention (eg, psychiatric consultation, care coordination, or psychological therapy). Although clinic-based treatment is often the cornerstone of care for people, physiological monitoring and physical health checks are a core part of treatment. In addition, data gathered by mHealth devices potentially allow for a more detailed, frequent, and cost-effective assessment process that may lead to the optimization of clinic-based appointments or home visits when required. The results of this review also highlight that mHealth devices can be integrated within multicomponent treatment programs or delivered as stand-alone interventions.
9	Kivelä et al [55], 2022	EMA for suicide research captures more variable aspects of suicide risk that are difficult to grasp through traditional retrospective questionnaires. The feasibility of the EMA method is encouraging, with the acceptance rate being 93%. Suicidal ideation exhibits substantial variability over time, often increasing or decreasing sharply within only a few hours in an individual. In addition to suicidal ideation itself, a number of its risk factors (such as negative affect, hopelessness, loneliness, burdensomeness, and thwarted belongingness) were also found to exhibit similar variability patterns and be associated with momentary ideation. Such variability in suicidal ideation may provide a more reliable index of suicide risk than the severity or duration of ideation alone, thus representing a promising marker for suicide risk. In the future, EMA can be made more reflective, introspective, and mindful of the experiences of patients through constant feedback.
10	Kulkarni et al [61], 2022	One of the most striking findings in the review was the variety of approaches used across the literature in terms of sensing (eg, active or passive) and the apps that were deployed for data collection. Although a small number of studies used existing frameworks and data sets, most of them developed various novel smartphone apps for their research. As such, this shows that there is a large scope for increased collaboration toward innovative approaches rather than the redevelopment of similar tools from scratch. The effective use of ML is essential for studies exploring the recognition of human activity by using digital systems using passive sensing for predicting user behavior. However, results show that there is a gap in the literature that could explore the scope for implementing more modern approaches in ML for smartphone sensing, but there has been a significant push to protect user privacy and provide users with greater control over their data. In addition, changes to privacy laws will also affect data collection, storage, and sharing practices.
11	Lo et al [50], 2020	Most studies had a limited amount of user activity data that were used to explore and understand the use of participants regarding their digital interventions. The researchers identified the associations between specific analytic measures and their respective outcomes. The recent publication time of the 49 papers included in this review suggests a growing interest in integrating analytics into mHealth evaluations. In fact, the observed diversity in objectives, sample sizes, and duration of exposure suggests that analytics can bring value to evaluations of efficacy, effectiveness, and feasibility studies using digital phenotyping. It is highly important to understand how to identify thresholds for high and low users as well as recognize the significance of correlations and relationships between analytic metrics and primary outcomes. It is also important to understand the characteristics and predictors of successful adoption of a consumer digital technology, which would ultimately provide a road map toward cost-effective and successful mHealth development for adolescents and young adults.
12	Marengo and Montag [49], 2020	The meta-analysis revealed that, on average, the accuracy of prediction of Big 5 personality scores of users through Facebook data mining was moderate. However, prediction accuracy was improved when the models included demographic variables and multiple types of digital footprints. There was also a general overlap in prediction accuracy among the traits of different users. It can be expected that the prediction accuracy might be more achievable in the future as large data sets become available (ie, the sample size is increased) and new types and forms of data are collected and mined for prediction purposes (eg, features extracted from visual data or location data). However, Facebook data are more feasible to obtain, and therefore, it is easy to infer individual characteristics unobtrusively. There is also an emerging need for more careful consideration of ethical challenges, and related sociopolitical consequences, of the use of extracted data—psychological targeting procedures might be used to target and manipulate people’s behaviors without the individuals being aware of it, ultimately reducing Hawthorne bias.
13	Melcher et al [11], 2020	Smartphone data streams not only revealed information about sleep, physical activities, and social interactions but also highlighted the potential to use these data to offer personalized and responsive mental health care for college students. The heterogeneity in the methods of the included studies in the review highlights the lack of standardized best practices and the nascency of this work. The most promising data streams included accelerometer, location, and screen use data. These studies generally focused on few mental health conditions (eg, anxiety) among college students. How all the data streams may be combined to gain a more holistic view of an individual’s mental health is still an open question.
14	Moore et al [45], 2016	The review indicated that assessing cognition through digital technology is feasible and generally accepted among research participants. Support is promising for the psychometric properties of mobile cognitive assessments, including good real‐world test-retest reliability and internal reliability as well as good convergent and discriminant validity with laboratory‐based assessments. Repeated mobile cognitive assessments could yield novel information on a broad range of applied research questions. The advantages of using mobile cognitive assessments can enhance the sensitivity of detecting subtle cognitive changes within the natural setting of a person’s home environment over the course of the study. Mobile cognitive assessments could also be used to provide a more valid characterization of cognitive difficulties over time, including real‐world individual baseline cognition before initiation of treatment, continuous collection of cognitive data over the course of treatment, and posttreatment cognition. Mobile cognitive assessments can be embedded within EMA protocols and also be integrated with sensor-based technology (eg, wearable devices). This will allow for the conduct of a systematic study of the effects of contextual and time-varying influences on real-time cognition and cognitive rehabilitation efforts.
15	Seppälä et al [58], 2019	This systematic review explored the use of data obtained from mobile phones and wearable sensors to support therapeutic intervention for psychiatric disorders or symptoms. There was high variability in participant selection criteria, investigation protocols, and data processing techniques, thus lacking generalizability in the associations between sensor-based data and clinical assessment. Building on the results of this review, m-RESIST^h^ set up a framework to create a CDSS^i^ based on a mobile therapeutic intervention for patients with schizophrenia. The CDSS is designed to provide users with the necessary information to support health-related and clinical decision-making. The system uses available data sources to assess the patient’s condition using decision algorithms and, as a result, classify the clinical condition to provide clinical and lifestyle recommendations. The CDSS starts with a training period of 2 weeks during which sensor-based data are collected, without activation of further system actions, to assess the patient’s baseline. Once trained, the system monitors the changes against the baseline.
16	Sequeira et al [56], 2020	Most of the evidence involved smartphone apps, with very few studies using actigraphy. Mobile and wearable devices captured a variety of data, including unobtrusive passive analytics; movement and light data; and physical and mental health data, including depressive symptom monitoring. Most studies also examined feasibility. Adolescents positively accepted mobile tracking for depression across the reviewed studies. The degree of accuracy in profiling mood variability can be enhanced by mobile technologies and data from EMA as well as components such as location tracking, call logs, and light sensors, allowing for a more thorough digital phenotyping of depression. All studies varied in their predicted outcomes, comparing model performance in a comprehensive way.
17	Thunnissen et al [60], 2021	The diverse possibilities of using EMA are underlined by the various EMA applications that were found for studying youth psychopathology, capturing a broad age range; several types of psychopathology; and different EMA procedures with regard to format, contingency, intensity, and duration. Potential gaps include the use of event-based EMA or parent-reported EMA across the types of psychopathology. Considering our findings regarding compliance, EMA methods seem generally feasible in various populations of youth with psychopathology. In addition, EMA appeared to provide measures with adequate construct validity, as indicated by findings of associations with related retrospective assessments.
18	Trifan et al [46], 2019	Smartphones were used in multiple health-related scenarios, such as physical activity (35/118, 29.7%) and mental health (33/118, 28%). Accelerometers and GPS were 2 of the most used sensors in smartphones for collecting data from which the health status or well-being of its users was inferred. Among the 118 selected papers, approximately 50 studies collected data from only 1 sensor, whereas most of the studies analyzed accelerometers to detect physical activity. More than half of the studies developed their system for Android smartphones. There is a lack of correlation between smartphone-generated outcomes and clinical knowledge. It should be noted that, among studies that aimed to monitor diseases, most of them targeted a specific population.
19	Versluis et al [59], 2016	In the within-subject studies (n=1008), a significant medium effect size of EMI^j^ on mental health outcomes of 0.58 was found. The estimated effect size did not differ or outcome type (ie, anxiety, depression, perceived stress, acceptance, relaxation, and quality of life), although no significant effect was found for relaxation. Moderation analysis suggested that the effect on mental health was 62% larger when the EMI was part of a treatment package that included the support of a mental health professional compared with EMI alone. New research studies should direct their efforts to assess the active features of an EMI that offer great potential for providing easy and cost-effective strategies to improve mental health and also prove to be positive for the psychological well-being of the population.
20	Weizenbaum et al [48], 2020	To date, real-time cognitive data collection via EMA has the potential to inform diagnosis and intervention efforts by capturing fluctuations in the cognition of the patients. Results of the studies suggest that both internal (eg, affect and motivation) and external (eg, time of day, surrounding noise, and recent activity) factors can affect cognitive performance at any given moment. EMA would give rise to preventative interventions for mitigating cognitive dysfunction and decline—improving clinical care—and the possibility of using open platforms in a variety of research and clinical settings.
21	Zarate et al [44], 2022	This research concerning the digital footprint of depression tends to be recent and skewed toward adult samples and high-income countries as compared with their counterparts. In addition, there was a significant focus on assessing mood or affect compared with other clusters of symptoms related to depression (eg, psychomotor activity, sleep quality, and social functioning). The field is expanding rapidly given that most included studies were published within the last 4 years. Future research should assess different symptoms of depressive disorders in children, adolescents, and young adults and investigate populations of depression from low- and middle-income countries, exploring and evaluating the mental health information obtained from social media content with a broader perspective.

^a^EMA: ecological momentary assessment.

^b^ML: machine learning.

^c^fMRI: functional magnetic resonance imaging.

^d^RDoC: Research Domain Criteria.

^e^EEG: electroencephalogram.

^f^BOLD: blood-oxygen-level-dependent.

^g^mHealth: mobile health.

^h^m-RESIST: mobile therapeutic attention for patients with treatment-resistant schizophrenia.

^i^CDSS: clinical decision support system.

^j^EMI: ecological momentary intervention.

### Statistical Analyses of EMA and Trace Data

Data analysis of longitudinal data collected intensively is challenging. Of the 21 included reviews, only 10 (47%) reported additional information on the statistical analyses used in the included studies. In particular, studies included in the reviews mainly implied descriptive statistics, correlations (to examine the statistically significant associations between features extracted from smartphones and well-being behaviors), pretest-posttest comparisons [54], or just exploratory approaches [61] to evaluate the data collection capacities of the apps. Similarly, reviews on interventions aimed to examine how different interventions were related to user well-being and engagement using hypothesis testing approaches [50]. In addition, some studies used multilevel or mixed models (fixed and random effects) that accounted for the nested structure of EMA data (eg, repeated measurements of participants over multiple days) [51]. However, only a few studies explored within-person fluctuations to predict mental well-being in youth [60].

The following reviews included machine learning–based approaches. In particular, Benoit et al [53] summarized machine learning techniques in the context of psychosis. They reported that, of 51 passive digital phenotyping studies, 16 (31%) included classification-based machine learning (to predict discrete class membership), regression-based machine learning (to predict continuous outcomes), and combined classification- and regression-based clustering analyses. In addition, in the case of Cornet and Holden [42], studies with >3 sensors relied on machine learning predictions, and the data were correlated with validation measures to test the validity of the interpretations or predictions. Built machine learning models were tested to predict user behavior (eg, location and activity to predict drinking episodes), sometimes even while the study was still running [61]. In addition, different models allowing for the recognition of ongoing activity were sometimes compared, for example, to distinguish among different physical activities such as walking, walking upstairs, walking downstairs, sitting, standing, and lying down [61]. Finally, 2 reviews included a meta-analytic summary of the results [49,59]

### Data Validation

A critical point is that only 43% (9/21) of the reviews reported the presence of control groups in the included studies [43,45,47,50,52,55,57,59,60] to compare the effectiveness of interventions or mobile assessments with respect to standard interventions or methods of data collection. In addition, only 52% (11/21) of the reviews reported that data in the included studies were validated using traditional assessments with standardized tools, referred to as “ground truth” [42]. The latter usually included validated questionnaires; medical records; study staff; and interviews administered by a clinician, such as the Structured Clinical Interview for the Diagnostic and Statistical Manual of Mental Disorders, Fourth Edition, or the predictive power of digital assessments to effectively differentiate participants with mental illnesses from healthy controls [53,54]. However, when present, ground truth data were usually implied just in half of the included studies [46]. A review reported a list of different validation tools adopted (mainly for depressive symptoms), including the Young Mania Rating Scale, Hamilton Rating Scale for Depression, Beck Depression Inventory, Patient Health Questionnaire, Children’s Depression Inventory, Center for Epidemiological Studies Depression Scale, Children’s Hassles Scale, Stress-Reactive Rumination Scale, Structured Clinical Interview, Scale for Cognitive Assessment in Neuropsychiatry, Mini-International Neuropsychiatric Interview, and Positive and Negative Syndrome Scale [43,53,56]. The RDoC framework was also used for cross-validation [57], whereas in some cases, results from cognitive tests were supported by traditional neuropsychological tests [45]. Interestingly, one review showed that EMA measures had a higher predictive power for suicidal ideation and more severe cases of ideation were identified through EMA than through retrospective questionnaires. Hence, EMA might increase the potential of detecting momentary and fleeting negative mood and suicidal ideation [55].

### Quality Assessment and Participant Adherence

**Figure 2:**
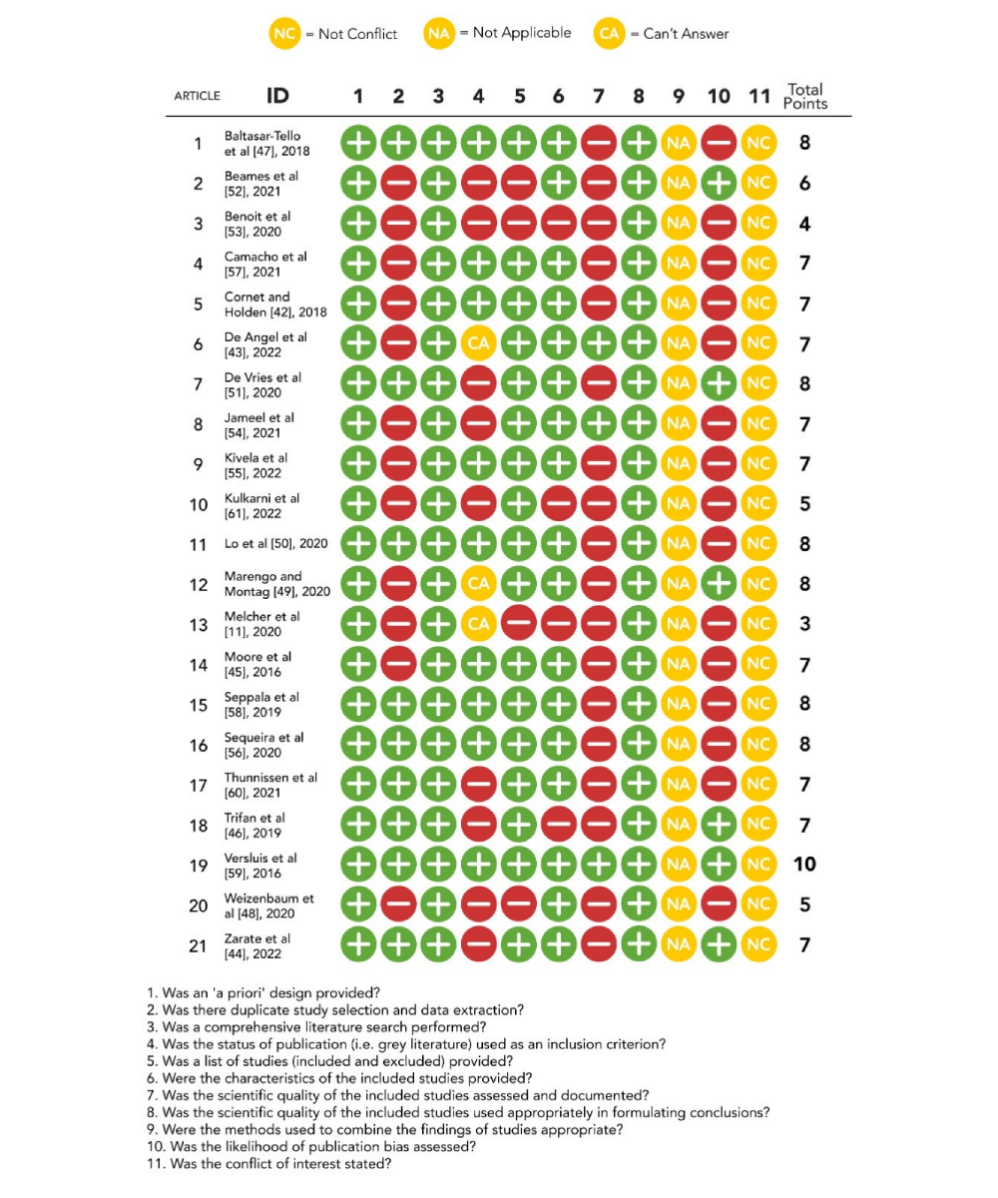
Summary of the quality assessment of the included reviews using the AMSTAR (Assessment of Multiple Systematic Reviews) [11,46-64,66].

The results of the quality assessment of the 21 systematic reviews (Figure 2 [11,42-61]) using the AMSTAR checklist showed that most of them were of medium quality (13/21, 62% received a score of ≤7 points). The average quality was 6.8 (SD 1.55). Only 33% (7/21) of the reviews could be considered to be of high quality. Of note, only 14% (3/21) of the reviews carried out quality assessment of the included studies, whereas 29% (6/21) assessed the presence of publication bias. Most reviews (17/21, 81%) effectively reported how they conducted the systematic search by indicating the inclusion and exclusion criteria. None of the 21 included reviews acknowledged a conflict of interest. In addition, all reviews (21/21, 100%) combined the scientific quality of the included studies appropriately when formulating conclusions.

In addition, only 38% (8/21) of the reviews presented reports on compliance rates, which largely varied and were not available in all the included studies. For example, de Vries et al [51] reported that only 47% of the included studies showed information on compliance rates with EMAs, and usually, compliance rates ranged between 43% and 90% [45,51,54,55]. However, in some cases, it was not possible to calculate adherence rates because of EMA using event-based prompts [45]. Another review showed an average compliance rate of 55.4% with EMA [52]. In addition, compliance rates have been described as being lower in clinical groups and patients of psychiatry and declining over time [55]—probably because of the fatigue effect. However, they did not vary among studies that used one or multiple daily assessments. Moreover, participants seemed to prefer answering in the afternoons and on weekends, and their response times decreased over time [55].

### Summary of Narrative Reviews

The included narrative reviews [10,66-73] mainly focused on the (1) discussion of the phenotyping problem, (2) importance of using an existing framework such as the RDoC matrix, and (3) new possibilities such as the use of digital phenotyping in the context of problematic digital media use, and they also (4) gave some practical examples from big studies.

First, it was underlined that large-scale phenotyping could play a substantial role in advancing biomedical sciences, especially as it allows for overcoming the ecological fallacy problem by collecting data in naturalistic settings. However, the inability to precisely specify phenotypes (ie, the observed manifestations of behaviors reflecting underlying biological mechanisms and genetic predispositions) is still an obstacle. The phenotyping problem is especially critical in psychiatry and neurology, where specific markers are desperately needed but individuals with these conditions may be unable to provide accurate self-reports. Social, behavioral, and cognitive phenotypes are challenging as they depend on temporal and contextual factors. Hence, digital phenotyping is promising as it collects data in a continuous rather than discrete period [10].

However, although promising, intensively collected data require new theoretical and conceptualization structures. It is possible to think about three steps: (1) defining appropriate phenotypes and related data to collect, (2) acquisition of continuous data and storage to permit large-scale and rapid analysis, and (3) processing data with specific analytical purposes [70]. For example, Torous et al [67] recommended using the RDoC criteria, launched in 2011 by the National Institute of Mental Health, as an alternative to symptom classification. The framework is a useful road map to show the state of the art and how future research should be framed to facilitate tailored medicine and interventions in mental health care. The goal is to quantify illnesses beyond patients’ self-reports and study them through different constructs measured across a spectrum of units of analysis, ranging from genes to behaviors. Through mobile tracing and assessment, the RDoC framework can potentially extend measurement to the general population with preclinical and nonobservable symptoms. By doing so, the RDoC-guided data collection can lead to the creation of transdiagnostic phenotypes. Hence, combining the RDoC framework and digital phenotyping could be a unique opportunity for psychiatric research to investigate new aspects of pathology that were largely unapproachable. Similarly, digital phenotyping may be used to conduct more extensive genetic, molecular, cellular, and neuroimaging investigations on specific mental health disorders.

EMAs and trace data could also be more or less valuable depending on the life span period investigated [72]. For example, 3 life span transitions are marked by smartphone use and increased psychopathology risk: the perinatal period, adolescence, and emerging adulthood. During these periods, gathering rich and objective information about emotion dysregulation and risk behaviors could be extremely difficult. However, passive monitoring, as a relatively cheap and unobtrusive type of data collection, can be an excellent opportunity to deepen the knowledge of the occurring emotion regulation or dysregulation processes. In particular, studying problematic digital media use, including social media, is a promising field, especially if coupled with sleep and mobility data. In addition, in the review by Montag and Rumpf [71], it was extensively described how digital phenotyping and mobile sensing from an interdisciplinary perspective rooted in psychoinformatics can be used as a tool to improve, prevent, intervene, treat, and care for web-based addictive behaviors and internet use disorder, considered an umbrella term for the different ways of overuse of web content. Opportunities rely on psychoeducation and blended approaches and models of care, allowing for the mapping of behaviors and tailored feedback and interventions. According to the review, digital interventions would bring about significant results during the first less intensive steps and aftercare.

Dunster et al [69] reported some examples from population-based studies such as the UK Biobank, the National Health and Nutrition Examination Survey in the United States, and the Health Family Study of Affective Spectrum Disorders, which tried to understand the directional associations among psychological states, sleep, and physical activity in people with bipolar disorder. For the authors, the simultaneous administration of instruments capturing subjective symptoms such as mood and cognition and objective information through passive sensing is recommended as passive monitoring would otherwise lack contextual information on variables that may play a role in everyday activities. In addition, physiological and biological data such as melatonin, cortisol, and heart rate, along with actigraphy and EMA, would further provide additional insights into the biological and physiological influence on mental disorders across the life span.

Finally, the narrative reviews pointed out how using mobile devices to collect data works for a short time and requires minimum effort from the user. However, immediate benefits should be shown to the user; otherwise, the risk of dropout increases [10]. Validation is still critical, so digital researchers are encouraged to simultaneously gather daily life self-reports (ie, ecological assessment) [71]. The most valid variables are likely smartphone-based assessment of problematic internet or social media use and sleep disturbance, followed by derivations of mobility, sociality, and phone activity. Finally, a limitation of the passive monitoring of markers is their complexity and bidirectional relationships with psychological states. For example, several researchers have disputed the degree to which sleep issues predispose one to regulatory problems or whether sleep problems result from emotion dysregulation. Researchers must consider the assessment of causality and increase the use of intensive longitudinal approaches with well-validated multilevel measurements. Designs are expected to be easier with passive monitoring, but it is difficult to unscramble the extent to which these measures index state- or trait-level emotion dysregulation [72].

## Discussion

### Principal Findings

This umbrella review summarizes 30 systematic and narrative reviews on mobile assessments and interventions using EMA and trace data in the context of mental health in youth identified through a systematic search and a hand search using the same keywords in January 2022 and updated in July 2022. Overall, a mobile approach seems generally feasible for various young populations with and without psychopathological symptoms—including the assessment and monitoring of mental health problems (especially depressive and psychotic symptoms), mental well-being, cognition, and personality in different contexts—using different data sources. For example, mobile assessments were frequently coupled with wearable devices but also tools such as neuroimaging and social media data, and interventions are fast moving to deliver tailored feedback.

The main results of the included reviews showed that, from a research viewpoint, collecting EMA and trace data allows for the assessment of momentary ill- and well-being in relation to different lifestyle factors (including media use, diet, sleep, ongoing pharmacological treatment, and cognitions) and also for the investigation of underlying cognitive processes in a way that is not possible using traditional methods. EMAs can detect cognitive biases such as recalling and generalizing negative memories in people with mood disorders, the daily fluctuations of suicide ideation, and the momentary awareness of one’s mood and affect. Daily monitoring is particularly important in the early stages of mental illnesses with low predictable trajectories that might become chronic, such as in the case of psychotic symptoms, schizophrenia, and bipolar disorder. By doing so, EMAs pave the way to delineate cognitive markers that can predict future outcomes (eg, the variability in suicide ideation), thus increasing symptom predictability—including their intensification and remission—over time. Moreover, with the collection of trace data, smartphone passive sensing has been described as more accurate and less intrusive, leading to the concept of “minimally disruptive medicine.” By doing so, passive sensing allows for the overcoming of the Hawthorne effect, defined as a “change in behavior as a response to observation and assessment” [74]. However, correlations between metrics and primary outcomes should be consistently recognized. Many studies still focus on one sensor (mainly GPS and accelerometers), and more research should test the best combination of data that can successfully predict a mental health outcome. To note, to delineate “the best combination,” a parsimonious approach should be followed in detecting effective internal (eg, affects, cognitions, and behaviors) and external (eg, social environment, time of day, and location) predictors of long-term outcomes by weighing the quality more than the quantity of collected data. This fine-grained overview of mental health symptoms would eventually allow for a detailed mapping of symptoms in existing frameworks such as the RDoC. In addition, more mental health outcomes and conditions should be investigated, ranging from serious mental illnesses to overarching concepts of well-being such as thriving and flourishing. By doing so, these approaches can be used in public health at large to monitor mental health changes and promotion in different contexts and periods and even in emergency situations.

**Figure 3:**
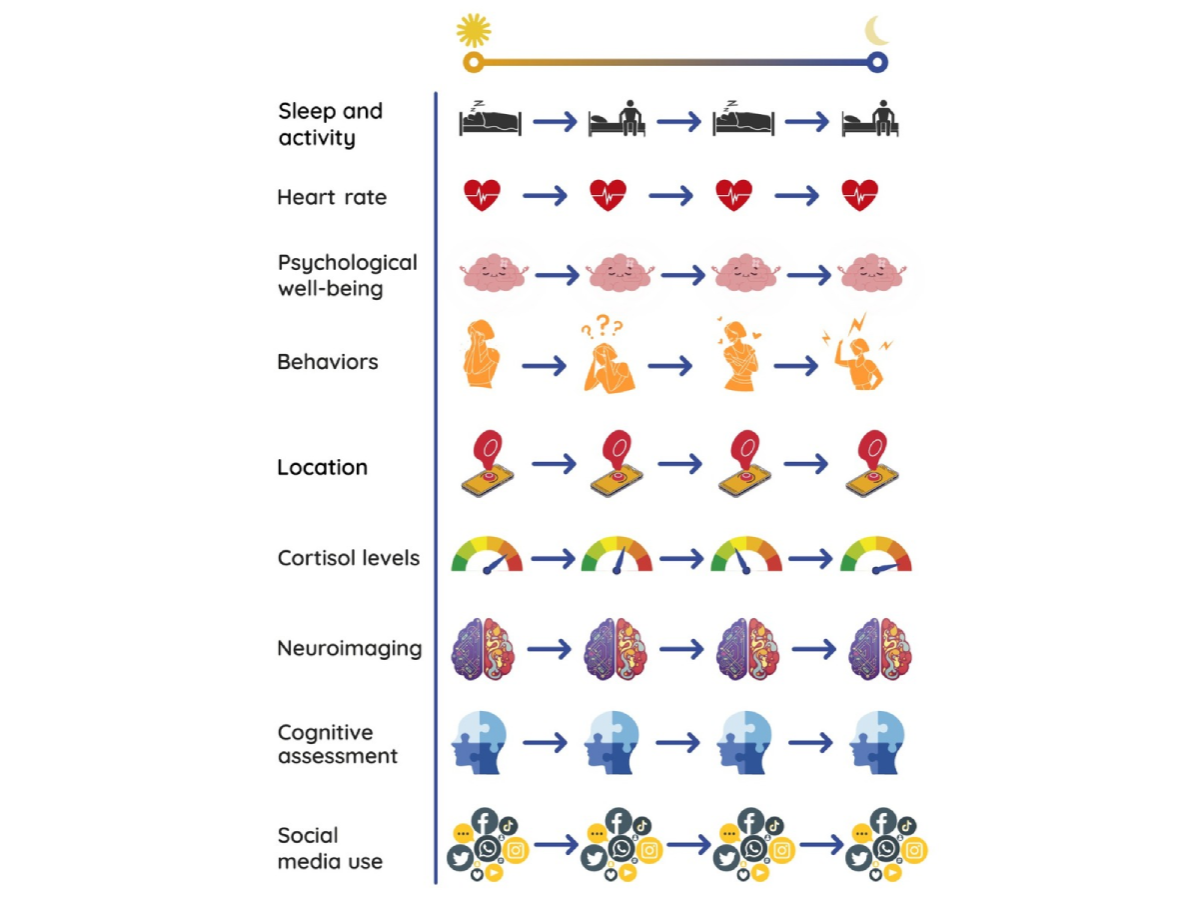
An overview of the types of data collected through mobile apps.

The included reviews also reported how to translate these research opportunities into the clinical setting. Interventions using EMA and trace data should consider that, by merely asking participants to report their current affect, their affect awareness will augment, and this process is already helpful in providing the foundations for good coping skills. In addition, the possibility of detecting subtle cognitive processes would also allow for better monitoring of the progress of a treatment, including pre- and postintervention differences. The collection of passive sensing data would improve just-in-time processing and personalized feedback during digital interventions in a tailored way. In addition, treatments involving EMIs and trace data have been described as particularly effective in combination with traditional support services received from mental health professionals (as part of a multicomponent treatment), bridging the gap between the clinical setting and patients’ everyday lives. Indeed, when digital tools can be integrated with ongoing treatment, they can optimize clinical-based appointments and the collection of information. It would even be possible to create clinical decision support systems based on collected data [58], based on machine learning algorithms, to classify conditions and provide lifestyle recommendations. However, these approaches can also be used as stand-alone interventions, for example, as support services for college students helping them with stress coping, academic-related anxiety, time management, attention problems, and social well-being. A visual summary of the results is presented in Figure 3.

In general, EMAs and trace data in the context of mental health assessment and intervention are promising tools and reasonable alternatives to existing traditional assessment and intervention protocols. However, this approach also has disadvantages, such as the absence of theoretical frameworks, the difficulty in data analysis, the poor availability of cross-validated data, and the difficulties in appropriately assessing the quality of the studies because of the lack of standards. In the following section, we summarize the results in terms of opportunities and limitations to guide future research and the development of new tools and interventions in the context of digitalized mental health care.

### Opportunities and Limitations of Mobile Assessments

#### Opportunities

A great opportunity for mobile assessments and interventions is the possibility of using them in LMICs as there is a significant care gap. Barriers to receiving mental health treatment include cultural interpretations of mental illness and lack of awareness and health care services for mental health. In addition, stigma and discrimination are barriers to even accessing services and recovering from mental illness [75]. Hence, allowing individuals to access mobile mental health care services in communities where there is no other available option would give them the opportunity to receive treatment in a safe and confidential way [75]. In line with a review on digital mental health care in LMICs [75], “mobile phone applications, if designed and tested rigorously, may provide a means for treating and managing a range of mental illnesses...in lower resource settings,” especially considering that approximately 80% of people with mental health issues reside in these countries but 90% of them do not have access to evidence-based treatments [7]. A review and meta-analysis [76] of 22 studies on digital interventions in LMICs showed that digital psychological interventions are moderately effective with respect to control interventions; however, high heterogeneity in the studies’ designs, settings, and participants calls for further studies. In addition, the included studies mainly focused on young adult samples, and no specific information was included regarding the use of EMAs and trace data in the context of web-based interventions. Moreover, it is important to acknowledge the barriers to conducting research in these areas. First, significant gaps in internet access should be addressed [77]—the global digital divide is large and mirrors economic inequalities, and even in higher-income countries, some populations (eg, those who live in rural areas and those with low levels of education) struggle to access the internet. In addition, a gender digital divide should be considered—women from LMICs have a lower probability of accessing the internet (first-level digital divide) but also show lower digital literacy levels than men (second-level digital divide) [78]. Hence, to roll out and implement promising digital mental health services in LMICs, it is crucial to highlight to policy makers and decision makers in general how technological development and improvement of mental health care services align. Moreover, health care workers should be involved in cocreating and designing digital interventions that are contextually relevant and easy to integrate into the existing community mental health services. In general, conducting studies based on mobile devices has the potential to reach younger people—those who are more familiar with new technologies—to participate in research and treatments. Hence, future studies should fill this gap as there is great potential for improving mental well-being.

In addition, the success of EMAs and trace data in psychology, psychiatry, public health, and related disciplines relies on theories—the inclusion of theories provides a stronger explanation for the findings and aids in data analyses. However, few attempts have been made so far. This requires the integration of multimodal data [79]. For example, a specific attempt for theory-driven research has been made with the use of the RDoC framework, which allows for elaborating more on the endophenotypes of mental disorders instead of relying on classic diagnoses based on the International Classification of Diseases, 11th Revision, or the Diagnostic and Statistical Manual of Mental Disorders, Fifth Edition. Specific symptoms of the RDoC subdomains can be expressed in diverse ways and domains, which would lay the groundwork for integrating data from different sources.

This umbrella review clearly shows how mobile apps for mental health care mainly focus on ill-being outcomes, such as general mental health problems, depression, anxiety, internet use disorder, and psychosis. However, as initially defined by the WHO, health is a “state of complete physical, mental and social well-being and not merely the absence of disease or infirmity” [80]. In addition, as established by positive psychology, “people also care about being happy, having a sense of meaning and purpose, being a good person, and having good relationships” [81]. It is crucial to consider that mental health includes a complex continuum ranging from ill-being to an optimal state of well-being. In total, 2 distinct yet related traditions of research on well-being should now be considered [82]. The hedonic perspective focuses on subjective well-being (SWB), defined as the intersection of life satisfaction and positive and negative mood, which eventually determine happiness. The eudaimonic view focuses on psychological well-being (PWB), defined as a state beyond just happiness as it includes different dimensions, from personal growth to positive relationships [83]. SWB and PWB are positively related to each other [84-88], linked to greater productivity at work, better health [81,82,89-91], lower levels of depression and distress, and a healthier immune system [92]. Mobile interventions based on positive psychology are still fairly nascent; however, conclusions from offline trials are promising [93-95]. Only 1 month of an intervention based on positive psychology showed effect sizes from small to moderate for mental SWB, PWB, and depression [93]. In addition, the effects persisted for up to 6 months. Thus, self-help interventions based on positive psychology “can serve as cost-effective mental health promotion tools to reach large target groups which may not otherwise be reached” [93] and possibly have a major impact on the population’s well-being. This might help create (mobile) interventions to promote well-being if we know what makes people happy. In addition, the findings can potentially inform policy makers and be applied to society at a larger scale. Indeed, recommendations, interventions, feedback, and reminders can be integrated into mHealth apps to provide users with useful information about their status and suggestions on how to improve their current situation. Smartphones and the use of advanced statistical models such as machine learning allow for the creation of personal profiles and conveying personalized support.

In addition, further studies should focus on how mobile phenotyping might be a useful way to improve self-efficacy and self-awareness, including affective awareness, and bridge the gap between 2 therapeutic sessions by facilitating the implementation of coping strategies. Hence, it is more likely that EMAs and trace data would be used as embedded training into other forms of therapy and clinical assessments rather than completely replacing them. Indeed, by allying the therapeutic dyad with mobile assessments, new opportunities for shared decision-making and supporting patients can be generated without obstructing the clinical workflow. EMAs and trace data would facilitate evidence-based and personalized care and create new insights into patient symptoms and outcomes to manage care [96]. In particular, the use of EMIs can serve to enhance the self-management of mental health problems. Of note, these interventions can be tailored to the person and be executed in real time using mobile devices.

Finally, the use of mobile assessments eliminates the need to recruit participants in offline settings for both assessments and interventions, especially for the general population. Hence, the possibility of recruiting participants via web (eg, through social media) allows for reaching underrepresented populations in research, such as minority groups often omitted in biomedical research [97]. In addition, web-based recruitment, assessment, and intervention allow for the exploration of complex psychological and social issues, such as addiction to substances; infectious diseases; and mental health problems in the context of chronic health conditions such as HIV, diabetes, cardiovascular disease, and cancer [97]. In addition, as reported by a previous review of EMA studies in youth [98], mobile assessments with EMAs and trace data can be conducted at young ages, although it is important to adapt features and protocols to different age ranges and backgrounds.

#### Limitations

In general, stemming from this umbrella review, it is hard to conclude whether digital assessments and interventions that include EMAs and trace data are more reliable and effective for monitoring and treating mental disorders than others because of the disproportionate number of studies comparing these approaches with classic ones (eg, face-to-face or web-based therapy and traditional self-report questionnaires). For example, according to a meta-analysis of digital interventions in children and adolescents with chronic medical conditions [99], the effect sizes of digital versus control conditions were small (or moderate) or not statistically significant. Another meta-review suggested that methodological limitations impeded drawing decisive conclusions from the included reviews on the efficacy of digital health interventions, including computer-assisted therapy, smartphone apps, and wearable technologies, for children and young people [100]. Similarly, a systematic meta-review [101] on the use of mobile phone–based interventions, including smartphone apps with and without additional support, meditation apps, SMS text message–based interventions, and general mobile phone–based interventions for mental health problems, failed to find convincing evidence of efficacy. The authors reported that the magnitude of the effects diminished as the comparison conditions became more rigorous. One way to improve our understanding of data collection, analysis, and modeling would be to use existing available data sets for secondary data analysis (eg, StudentLife) of sensor data, EMA data, survey responses, and others. For example, owing to the StudentLife data set, it was possible to explore how objective smartphone sensor data correlated with mental health and grade point average scores [102,103]. This approach would allow for greater transparency and the replication of analyses and results and generate new research questions related to mental health for future studies. Hence, although the results of this umbrella review are rather explorative, it is now crucial to investigate how EMAs and trace data can improve diagnosis and treatment over and above traditional methods (including web-based therapies).

The previous point is mainly related to the general drawbacks of digital phenotyping—mobile approaches to improve mental health should demonstrate their value not only in clinical effectiveness but also in the public health sector by tracing health at a larger scale and in different communities. The fact that information on data validation was mostly lacking demonstrates that we still do not know whether better measurement alone and multimodal data confer better outcomes. To go “from better data to better care,” new collected information should be able to make more accurate predictions, improve clinical decision-making and efficiency, and help in reaching the desired outcomes [104]. In addition, many studies focused more on “physical” symptoms, such as sleeping habits, physical activity, and movement, and fewer studies measured mood fluctuations and psychological nuances of well-being (eg, mood swings). In addition, the studies in the included reviews did not report data on vulnerable populations such as participants from low socioeconomic backgrounds, racial or ethnic minority groups, or LMICs. In addition, owing to the heterogeneity of the included reviews and the fact that only 29% (6/21) of the reviews reported more detailed information on age, it was difficult to create clear cutoffs for age. Thus, these results further fuel the problem of “data absenteeism,” described as “the absence of data in our studies from groups experiencing social vulnerability—whether by class, race or ethnicity, or geography—in sufficient quality and quantity” [105].

Another important area demanding attention is comprehensive reporting on the quality of EMA studies, including design decisions and features of data collection and participant adherence. Although rarely conducted in the included reviews, detailed reporting is necessary to properly understand the results and develop data-driven and best practice recommendations. Studies should report more on training procedures, incentives, and adherence rates to let future studies use this information. For example, future research should focus on how to maintain adherence—the individual needs to be convinced that the collected data will not produce any conflicts but will provide advantages such as mental health promotion, early detection of a mental health condition, monitoring of mental health difficulties, and interventions. In addition, specific future attention should be paid to privacy concerns depending on new legislation (eg, the General Data Protection Regulation). Moreover, several ethical issues should be addressed in developing new studies, including digital phenotyping in mental health; these aspects should tackle, for example, privacy, transparency, consent, accountability, and fairness [106]. In addition, users should be able to understand how their raw data will be analyzed to extrapolate information on mental health. At the same time, requirements and guidelines for the realization of digital behavioral monitoring platforms should be followed, including careful data isolation measures [107]. In this regard, it is highly recommended to implement acceptance-facilitating interventions—with clear information on benefits and risks—and improve users’ experience and acceptance of digital phenotyping and artificial intelligence applications [25]. Future studies should also explore the issue of ensuring trust and highlighting user benefits and consider which features embedded in apps would maximize the outcome in terms of data collected. For example, young people might prefer the presence of videos, limited text, ability to personalize and connect with others, and options to receive reminders [108]. Finally, guidelines should be developed to standardize procedures and assessments and test them in large samples. For example, a starting point might be to follow an ethics checklist to promote an appropriate digital research design using digital health technology in the context of psychiatry [109].

### Conclusions

To conclude, this umbrella review suggests that we are moving toward a “digital” shift in the mental health care paradigm [110]. Future studies should follow the aforementioned opportunities by using this approach in different settings—especially in LMICs—and modeling other psychological variables such as positive well-being and happiness. It is indeed exciting to think about how digital phenotyping and EMAs could be new tools to promote mental well-being. Mobile tools will soon allow for personalized interventions in real time, enabling people to modify their behaviors by receiving such feedback. However, although the digitalization of mental health assessments and interventions is now considered promising in overcoming existing treatment gaps, we should still look carefully at specific benefits and limitations of such an approach by considering different populations, mental health problems, and health care settings.

Although multimodal integration is appealing, more research should be conducted on how to analyze complex data. This step requires a strong multidisciplinary collaboration with computational sciences and statistics. So far, different efforts have been made to recognize multimodal patterns of sociability, physical activity, and home stay [111], combining digital phenotyping, EMAs, and machine learning to detect depressive symptoms [112]. As pointed out by Onnela and Rauch [110], “as digital phenotyping matures, it seems likely that the intellectual challenge will shift from data collection to data analysis and modeling.”

Further use of EMAs and trace data is in the context of studying “digital biomarkers” to model individual neurobiology from digital footprint patterns left from interactions with smartphones and social media [113]. This method would allow for the combination of genetic and neuroscientific information with data such as social media data to delineate underlying biology or neurobiology, thus augmenting our understanding of individual differences in various psychological processes, such as cognitive, motivational, and emotional processes, together with underlying neural mechanisms [114].

As stated by Jain et al [23], “whatever the pace and obstacles, the convergence of digital technologies and biology is an inevitability that is bound to change how we both understand and treat disease—and the concept of the digital phenotype is one that will only continue to grow in its significance.”

## Appendix

Multimedia Appendix 1 PRISMA (Preferred Reporting Items for Systematic Reviews and Meta-Analyses) checklist.

Multimedia Appendix 2 Search strategy.

Multimedia Appendix 3 Characteristics of included reviews.

## Abbreviations

AMSTAR: Assessment of Multiple Systematic Reviews
EMA: ecological momentary assessment
EMI: ecological momentary intervention
LMICs: low- or middle-income countries
mHealth: mobile health
MRI: magnetic resonance imaging
PRISMA: Preferred Reporting Items for Systematic Reviews and Meta-Analyses
PWB: psychological well-being
RDoC: Research Domain Criteria
SWB: subjective well-being
WHO: World Health Organization
