# Supplementary material for: The Use of Mobile Assessments for Monitoring Mental Health in Youth: Umbrella Review
Source: J Med Internet Res. 2023 Sep 19;25:e45540. doi: 10.2196/45540 (PMC10548333; doi:10.2196/45540)
Supplement: Multimedia Appendix 2 [file jmir_v25i1e45540_app2.docx]

**Multimedia Appendix 2**

**Search strategy**

digital phenotyp* OR digital phenotyping OR phenotyp* OR digital biomarker* OR digital health* OR digital profil* OR wearable* OR EMA* OR in-situ OR ecological momentary assess* OR ESM* OR experience sampling* OR ambulatory assess* OR trace data (TI)

AND

review* OR systematic* OR meta-analysis* OR metanalysis* OR synthesis* OR meta-review* OR analysis* (TI)

AND

young* OR adolesc* OR youth* OR adult* or teen* OR tween* OR child* (AB)
