# Supplementary material for: The Use of Mobile Assessments for Monitoring Mental Health in Youth: Umbrella Review
Source: J Med Internet Res. 2023 Sep 19;25:e45540. doi: 10.2196/45540 (PMC10548333; doi:10.2196/45540)
Supplement: Multimedia Appendix 3 [file jmir_v25i1e45540_app3.docx]

**Multimedia Appendix 3**

**Table S1. Included systematic reviews (n=21).** Legend. ID= Review’s first author. N=number of included studies. Sample= number of total participants included in the review. Age= C=children, A=Adolescents, Y=Young adults

| **ID** | **Title** | **Journal** | **Review’s aim** | **Included databases** | **Date of search** | **N** | **Sample** | **Age** | **Countries** |
| --- | --- | --- | --- | --- | --- | --- | --- | --- | --- |
| Baltasar-Tello 2018 | Ecological Momentary Assessment and Mood Disorders in Children and Adolescents: a Systematic Review | Current Psychiatry Reports | The aim of the present work is to review published data regarding Ecological Momentary Assessment (EMA) in children and adolescents with mood disorders and evaluate results following usefulness, feasibility, and acceptability criteria. | PsycInfo, PubMed, and Google Scholar | December 2017 | 13 | 2140 | C,A | NA |
| Beames 2021 | Prevention and early intervention of depression in young people: an integrated narrative review of affective awareness and Ecological Momentary Assessment | BMC Psychol | The aims of this review are to assess: (1) whether EMA increases affective awareness and reduces depression symptoms; (2) whether EMA can identify depression risk; and (3) stakeholder perspectives about affective awareness and using EMA in daily life, in and outside of the therapy context. | PsycInfo, PubMed, EMBASE, Google Scholar and CINAHL | July 2020 -August 2020 | 11 | 1263 | A,Y | USA, Australia, Belgium, and Netherlands. |
| Benoit 2020 | Systematic Review of Digital Phenotyping and Machine Learning in Psychosis Spectrum Illnesses | Harvard Review of Psychiatry | This review addresses techniques in digital phenotyping research successfully applied to passively collect smartphone data and to assess symptoms of clinical high risk, early course, chronic schizophrenia, psychosis, and bipolar disorder with a focus on machine-learning studies | PubMed, Web of Science, PsycInfo, Embase, and the Cochrane Central Register of Controlled Trials | Until September/October 2020 | 51 | NA | Y | USA, UK, Denmark, Austria, Australia, China, and South Korea. |
| Camacho 2021 | Advancing translational research through the interface of digital phenotyping and neuroimaging: A narrative review | Biomarkers in Neuropsychiatry | The authors synthesize the current evidence for interconnectedness of neuroimaging and smartphone data, with the goal of understanding both the research and clinical potential. Also, they organize studies by NIMH’s Research Domain Criteria (RDoC) and constructs with the goal of understanding progress in the context of a translational neuroscience framework to allow assessment of how digital measures are being used to bridge the neural circuitry and behavioral/self-reported domains. | PubMed and PsycInfo | June 2020 | 14 | 2065 | A | NA |
| Cornet 2018 | Systematic review of smartphone-based passive sensing for health and wellbeing | Journal of Biomedical Informatics | The aim of this review is to analyze published empirical literature on the use of smartphone-based passive sensing for health and wellbeing. | ACM Digital Library, PubMed, Web of Science | January 2017 | 35 | 835 | Y | USA, Europe, China, Korea, and Mexico. |
| De Angel 2022 | Digital health tools for the passive monitoring of depression: a systematic review of methods | npj Digital Medicine | The aim of this study is to review studies linking passive data from smartphone and wearable devices with depression and summarise key methodological aspects, to: (a) identify sources of heterogeneity and threats to reproducibility, and (b) identify leading digital signals for depression. | PubMed, PsychInfo and Embase | 2022 | 51 | 9303 | Y | USA, Australia, Brazil, Japan, South Korea, China, Canada, Netherlands, Switzerland, and Ireland. |
| De Vries 2020 | Smartphone-Based Ecological Momentary Assessment of Well-Being: A Systematic Review and Recommendations for Future Studies | Journal of Happiness Studies | The aim of this review is to analyze what are the used designs (context, schedule, sampling, Well-being (WB) measure, applications, smartphones and statistical analyses) in smartphone-based EMA studies to well-being. Also, to what extent is objective data, such as GPS or accelerometer data included in smartphone-based EMA well-being research? Is the response rate and compliance in smartphone-based EMA studies related to the design? What are the results of smartphone-based EMA studies with respect to well-being? Finally, what are the limitations in current smartphone-based EMA? | PubMed and Web of Science | Until September 2019 (Updated in August 2020) | 53 | 97,454 | A,Y | USA, Germany, UK, Canada, Australia, Spain, UK, Netherlands /Lithuania |
| Jameel 2021 | mHealth technology to assess, monitor and treat daily functioning difficulties in people with severe mental illness: A systematic review | Journal of Psychiatric Research | The aim of this systematic review is t synthesize the evidence base for applying and using mHealth to support functional recovery in people with severe mental illness (SMI); to evaluate the acceptability and feasibility of mHealth assessment, monitoring and interventions targeting daily functioning problems in people with SM; to assess the methodological quality of the available evidence, and to identify future directions for research. | Embase, PubMed and PsycInfo,Google Scholar and reference lists of retrieved papers hand-searched. | January 2020- December 2021 | 38 | 2262 | Y | USA, Portugal, and Germany. |
| Kivela 2022 | Don’t Miss the Moment: A Systematic Review of Ecological Momentary Assessment in Suicide Research | Frontiers in Digital Health | This systematic review was conducted to asssess EMA studies in suicide research to evaluate how EMA has been utilized (i.e., methodology, findings), and to assess the feasibility, validity and safety of EMA in the study of suicidal thoughts behaviors.. | Web of Science, PubMed | December 2021 | 45 | 3082 | A,Y | NA |
| Kulkarni 2022 | Opportunities for Smartphone Sensing in E-Health Research: A Narrative Review | Sensors | The main goal of this study is to understand the potential of smartphone sensing in healthcare to provide a full understanding of existing practice. | Google Scholar and PubMed | 2017- 2022 | 86 | NA | Y | NA |
| Lo 2020 | Surveying the Role of Analytics in Evaluating Digital Mental Health Interventions for Transition-Aged Youth: Scoping Review | JMIR MENTAL HEALTH | This review aimed to examine how usage data are collected and analyzed in evaluations of mental health mobile apps for transition-aged youth (15-29 years). | CINAHL, Embase, PubMed, PsycInfo, and Cochrane Central Register of Controlled Trial. | 2008-2018, Updated in June 2019 | 49 | NA | A,Y | USA, Australia, Germany, Canada, Ireland, Japan, Netherlands, New Zealand, Norway, Romania, Spain, Sweden, and UK. |
| Marengo 2020 | Digital Phenotyping of Big Five Personality Traits via Facebook Data Mining: A Meta-Analysis | Digital Psychology | This article analyzes studies performing predictions of Big Five personality traits based on Facebook data. | Scopus, ISI Web of Science, PubMed, and ProQuest | July 2018 | 21 | 438113 | Y | NA |
| Melcher 2020 | Digital phenotyping for mental health of college students: a clinical review | Evid Based Ment Health | This review aims to summarize the current practices in digital phenotyping for college student mental health in order to describe how digital phenotyping can best serve college students and to identify best practices. | Google Scholar | May 2020 | 25 | 2032 | Y | NA |
| Moore 2016 | Applications for self‐administered mobile cognitive assessments in clinical research: A systematic review | Int J Methods Psychiatr Res. | This systematic review assesses research studies that employed repeated mobile device‐based cognitive testing in clinical and non‐clinical populations to examine the feasibility of mobile cognitive assessments as well as summarizing the specific applications, research design, cognitive domains assessed, and measured reliability and construct validity in these studies. | PubMed | August 30, 2016. | 12 | 885 | A,Y | Europe and USA |
| Seppala 2019 | Mobile Phone and Wearable Sensor-Based mHealth Approaches for Psychiatric Disorders and Symptoms: Systematic Review | JMIR MENTAL HEALTH | The aim of this study is to systematically review original studies on sensor-based mHealth apps aimed at uncovering associations between sensor data and symptoms of psychiatric disorders in order to support the m-RESIST approach to assess effectiveness of behavioral monitoring in therapy. | Scopus, PubMed, Web of Science, and the Cochrane Central Register of Controlled Trials | September 2018 | 35 | 1996 | Y | NA |
| Sequeira 2020 | Mobile and wearable technology for monitoring depressive symptoms in children and adolescents: A scoping review | Journal of Affective Disorders | The aim of this study is to review mobile and wearable tools to characterize depression in children and/or adolescents. | PubMed, PsycInfo, Embase, LISTA, and CINAHL. | July 2018 (Updated Feb 2019) | 32 | NA | C,A | USA, Western Europe and Australia |
| Thunnissen 2021 | Youth Psychopathology in Daily Life: Systematically Reviewed Characteristics and Potentials of Ecological Momentary Assessment Applications | Child Psychiatry & Human Development | The aim of this study is to review the characteristics of current EMA applications for youth psychopatology and to provide synthesis of their potential in studying youth psychopathology in terms of feasibility and validity of EMA in youth with psychopathology. The study also describes studying the phenomenology of youth psychopathology and its correlates in daily life, including parent–child interactions, and using EMA in evaluating treatment outcomes. | PsycInfo and PubMed | April 2020 | 50 | 4012 | C,A | NA |
| Trifan 2019 | Passive Sensing of Health Outcomes Through Smartphones: Systematic Review of Current Solutions and Possible Limitations | JMIR Mhealth Uhealth | The aim of this study is to identify recent scientific studies that explored the passive use of smartphones for generating health- and wellbeing- related outcomes. Also, it explores users’ engagement and possible challenges in using such self-monitoring systems. | PubMed, IEEE Xplore, ACM Digital Library, and Scopus | 2018 | 118 | NA | Y | NA |
| Verslius 2016 | Changing Mental Health and Positive Psychological Well-Being Using Ecological Momentary Interventions:A Systematic Review and Meta-analysis | JOURNAL OF MEDICAL INTERNET RESEARCH | The aim of this study was to systematically assess and meta-analyze the effect of EMI on three highly prevalent mental health outcomes (anxiety, depression, and perceived stress) and positive psychological outcomes (eg, acceptance). | PsycInfo and Web of Science | Septemebr 2015 | 33 | 1301 | Y | Netherlands |
| Weizenbaum 2020 | Cognition in Context: Understanding the Everyday Predictors of Cognitive Performance in a New Era of Measurement | JMIR MHEALTH AND UHEALTH | This paper describes selected literature on contextual factors that examined how experimentally induced or self-reported contextual variables (ie, affect, motivation, time of day, environmental noise, physical activity, and social activity) related to tests of cognitive performance. the study also included papers that used mobile assessment of cognition. | -- | -- | 14 | 5277 | Y | NA |
| Zarate 2022 | Exploring the digital footprint of depression: a PRISMA systematic literature review of the empirical evidence | BMC Psychiatry | The aim of this study is to identify empirical research examining the use of digital phenotyping to study depression; to describe the different methods and technology employed; to integrate the evidence regarding the efficacy of digital data in the examination, diagnosis, and monitoring of depression and to clarify digital phenotyping definitions and digital mental health records terminology. | PsycInfo, PubMed and Scopus | 2021 | 118 | NA | A, Y, C | USA, Netherlands, Germany, Belgium, Canada, and Brazil |
|  |  |  |  |  |  |  |  |  |  |
|  |  |  |  |  |  |  |  |  |  |

**Table S2. Main characteristics of included systematic reviews (n=21).** Legend: Duration= Mean duration of included studies in weeks. Intensity= frequency of data collection per day. Mobile tool(s) used= smartphone/sensors/other. NA=Not Available

| **ID** | **Review on Tracing or Intervention** | **Duration** | **Intensity** | **Mobile tool(s)** | **Other tool(s)** | **Information on applications used** |
| --- | --- | --- | --- | --- | --- | --- |
| Baltasar-Tello 2018 | BOTH | 6 | NA | SMARTPHONES/OTHER | Actigraphy, fMRI, salivary samples, pupil dilatation and pupillary responses | Daybuilder, Mobile Mood Diary, ACT app (focusing on mood disorders) |
| Beames 2021 | BOTH | NA | NA | SMARTPHONES | CBT = cognitive behavioural therapy, STEP = Skills to Enhance Positivity Program | NA |
| Benoit 2020 | TRACING | 17 | NA | SMARTPHONES | Wearable sensors used: accelerometer/ gyroscope, heart rate, other. | 25 unique apps in 40 studies (with 12 studies not reporting app name used) were used to collect data |
| Camacho 2021 | TRACING/INTERVENTION/PROTOCOLS (Of the 14 articles that met inclusion criteria, five were identified as protocols. These future studies plan to utilize MRI and fMRI to gather neuroimaging.) | 5 | NA | SMARTPHONE/NEUROIMAGING | MRI, fMRI, EEG, and PET | Monsenso BeHapp Mindstrong BeHapp BiAffect Smartphone text messaging PsyMate (electronic portable device) Smartphone- based EMA StudentLife Smartphone- based EMA NeuroSence MovisensXS Smartphone- based EMA Unspecified Mobile App |
| Cornet 2018 | TRACING | 4 | NA | SMARTPHONES | NA | 31 studies (89%) used the Android operating system (OS), compared to two using Apple iOS (5.7%), and one using the now-defunct Symbian OS (2.9%) |
| De Angel 2022 | TRACING | 1 DAY-1 YEAR | 24 h of consecutive data | SMARTPHONES/WEREABLE DEVICES | Actigraphy-based wrist-worn devices including one Fitbit and a Microsoft band, as well as one pedometer and smartphones (both android and iPhone). | Actiwatch 4, Cambridge Technology Ltd, GENEActiv, Activinsights, UK, Motionlogger, Ambulatory Monitoring, Ardsley, NY, Empatica, Tempatilume® (Cebrasil, Inc. Brazil), Pedometer, Microsoft Band, Garmin,Actiwatch Mini Mitter Co. Inc. |
| De Vries 2020 | TRACING | 2 | Mean= 5 prompt/day (from 2 to 12) | SMARTPHONES/WEREABLE DEVICES | Biosensors, personal digital assistant (PDA), palm-top computer, hand held computer (additional accelerometer to wear on the arm, chest or hip, or heart rate meter) | The (data of the) Mappiness app (k = 5), the Well-being Science app (k = 2), SnackImpuls app (k = 2), the CalFit app and a specific movisensXS app were used in multiple publications to answer different research questions |
| Jameel 2021 | BOTH | 1 DAY-1 YEAR | Mean=10 times per day | SMARTPHONES/WEREABLE DEVICES | Participants must use an mHealth device (e.g. iPad, personal digital assistant, mobile phone technology, wearable or wireless devices) for assessment, monitoring or delivering an intervention (or at least in part e.g. blended with clinic based sessions and/or ongoing input from a clinician) | NA |
| Kivela 2022 | TRACING | 4 | The number of (scheduled) EMA prompts per day ranged from 1 to 11 (median = 5, n = 21) | SMARTPHONES | NA | NA |
| Kulkarni 2022 | TRACING | NA | NA | SMARTPHONES | NA | MovieSens XS, BeHapp, AWARE, RADAR—base, Beiwe (Both open-source and Software-as-a-Service (SaaS) framework for data collection and analysis), EARS (Initially open-source, now available as SaaS for data collection and analysis, Emotion Sense, GPS,, Gyroscope, microphone, battery level, Phone-call and text-message logs, Accelerometer, Ambient light. |
| Lo 2020 | INTERVENTION | 8 | NA | SMARTPHONES | NA | Classification using the WHO Classification of Digital Health Interventions revealed that several mental health apps (n=11) exhibited more than one function. Three features that were most commonly found among the evaluated interventions were targeted client communication (n=39), client-to-client communication (n=8), and on-demand information services (n=5). Apps included:  CATCH-IT  ACT-CL  AlcoholEdu  BASICS-Mobile  CALM  DEAL  eBody Project  E-couch  HORYZON  ibobbly  LEAP  MoodGYM  Panoply  PRIME  Reach Out Central (ROC)  Rebound  Sexunzipped  SIGMA  SilverCloud  SocialVille  SPARX-R  Student Bodies  StudentBodies  The Toolbox  ThinkFeelDo  ViBe |
| Marengo 2020 | TRACING | NA | NA | SMARTPHONES/OTHER | Facebook data, Apply Magic Sauce, IBM Watson Personality Insight. | MyPersonality data |
| Melcher 2020 | TRACING | 6 | EMAs from 1 to 8 times/day | SMARTPHONES/WEREABLE DEVICES | Wearable technology such as smartwatches and activity trackers | NA |
| Moore 2016 | TRACING | 1 | from 2 to 5 daily | SMARTPHONES/WEREABLE DEVICES (In general, studies conducted prior to 2009 used personal digital assistants (PDAs; n = 4) and studies after 2009 used a mobile phone (n = 8)) | Physiological monitoring (in one study portable bio signal recorder, ECG electrodes, and acceleration sensors were affixed to participants in the laboratory). | Each study reported developing these instruments “in house”, and none of the tests are publicly available in app stores |
| Seppala 2019 | TRACING | NA | NA | SMARTPHONES | NA | NA |
| Sequeira 2020 | TRACING | NA | NA | SMARTPHONES/WEREABLE DEVICES | Handheld computers, actigraphy, which are wristwatch like devices, belt-like actigraphy | Smartphone applications (apps) used included: Acer Liquid Z-200  Mobiletype, CopeSmart, PETE, SOLVD , eMate, iYouVU, daybuilder, and Studentlife |
| Thunnissen 2021 | TRACING | 2 | from 2 to 12 | SMARTPHONE/OTHER (Various formats were used to obtain data: phone calls (17 studies); applications installed on a personal digital assistant (PDA) (14 studies), smartphone (9 studies), or iPod touch (3 studies); diary booklets, online surveys | digital wristwatch or device; actigraph. One study also used vials to collect saliva samples for cortisol assessment, physiological variables measured by laboratory paradigms | NA |
| Trifan 2019 | TRACING | 4 | NA | SMARTPHONES | NA | Android and IOS, other app not mentioned. Of the selected papers, 56.7% (67/118) developed their system only for Android smartphones, 6 developed for both Android and IOS, and 45 did not provide any information about the chosen operating system. |
| Verslius 2016 | INTERVENTION | 7.5 | 2.80 training episodes per day (SD=2.12) ranging from 1 to 10 | SMARTPHONES/OTHER | 10 studies combined EMI with therapy, mCBT :mobile cognitive behavioral therapy; mIPT: mobile interpersonal psychotherapy; MP3: audio only condition;  Nnar: video only condition; Vnar:video narrative condition; VRMB: virtual reality and mobile condition with biofeedback; VRM:virtual reality with mobile condition. | NA |
| Weizenbaum 2020 | TRACING | NA | NA | SMARTPHONES/WEREABLE DEVICES | apple watch, PDA, | NA |
| Zarate 2022 | TRACING | NA | NA | SMARTPHONES/WEREABLE DEVICES | heart patch, wristwatch, digital ring | NA |

**Table S3**. **EMA, trace data, and psychological symptoms investigated in the included systematic reviews (n=21)**. NI= not included. NA= information not available.

| **ID** | **EMA or Trace data** | **Details on trace data** | **Details on EMA data** | **Psychological symptoms/Behaviors that the studies traced/intervened on.** | **Category of the conditions included** |
| --- | --- | --- | --- | --- | --- |
| Baltasar-Tello 2018 | EMA | NI | Telephone interviews conducted by trained professionals.  No published articles on mood disorders and EMA that followed a protocol using a web page or mobile app to perform the measurements. | Daily emotional dynamics of child and adolescent depression, familial aggregation and risk factors of mood disorders, co-rumination during interactions with peers and parents in adolescents with major depressive disorder (MDD), relationship between caffeine consumption and affect/sleep in youth with depression, assessment of the association between media use and major depressive disorder, examine within-day fluctuations in negative affect and relationship in self-focus, evaluation of treatment response in adolescents with MDD, anxiety, or comorbid MDD and anxiety. | Mood Disorders |
| Beames 2021 | EMA | NI | App-based EMA monitoring of various states to explore daily emotion dynamics, usually used as a measurement tool and intervention | EMA engagement predicted decreases in depression. Self‐monitoring, via EMA, increased emotional self‐ awareness, but had no effects on depression. Self‐monitoring, via EMA, had an indirect effect on depressive symptoms via emotional self‐ awareness. The direct effect was not significant EMA mood monitoring had no effect on affective experience or depressive symptoms EMA mood monitoring had no effect on affective experience or depressive symptoms | MDD and Mood. |
| Benoit 2020 | TRACE DATA | Phone sensors used: accelerometer/ gyroscope, GPS, microphone, ambient light, screen touches, heart rate, other; Phone analytics collected: charging status, battery status, screen unlock/ lock, screen on/off, call logs, cellular data use, text/SMS logs, Bluetooth connections, Wi-Fi, cell tower connections, apps used, keyboard use. | NI | Passively tracked behavior to predict aggregated scores of mental health in schizophrenia in relation to natural environmental variables and contexts (e.g. nature, daylight, urban). | Clinical high risk, early course, chronic schizophrenia, psychosis, and bipolar disorder. |
| Camacho 2021 | EMA/TRACE DATA | NA | The domain with the largest number of studies classified within it was the Negative Valence Systems with eight of the fourteen studies included. This is followed by a two-way tie between Positive Valence Systems and Social Processes with four of the fourteen studies included. Negative Valence Systems n = 8; Positive Valence Systems n = 4; Social Processes n = 4; Arousal and Regulatory Systems n = 3; Sensorimotor Systems n = 3 Cognitive Systems | Several of these studies will be gathering data on more than one population. Three studies will be focus on depression, two will study schizophrenia and Alzheimer’s Disease, one will examine obesity, and one will study bipolar disorder. Seven of the 14 articles that met inclusion criteria, were studies that monitored behavior. These included studies incorporated several types of neuroimaging including MRI, fMRI, EEG, and PET. The most widely used imaging tool among these studies was fMRI which was used across five studies. According to the Research Domain Criteria (RDoC) criteria, eight studies focused on negative valence systems, four on positive valence systems, four on social processes, three on arousal and regulatory systems, three on sensorimotor systems, and one on cognitive systems. | MDD and obesity, schizophrenia, bipolar disorder, |
| Cornet 2018 | TRACE DATA | Accelerometer, GPS, SMS patterns, call logs, Bluetooth, light sensor, browser history, microphone, light sensor, application usage, calendar. The most used physical sensors were the accelerometer (25 studies), Global Positioning System sensor (GPS; 22 studies), light sensor (10 studies), and microphone (9 studies). Studies also collected data on device analytics, including call logs (14 studies), device activity (defined as screen on/off and device on/off; 11 studies), and Short Message Service (SMS) patterns (frequency and/or recipients; 11 studies). most studies combined multiple sensors. |  | Mental health was the most common application domain for studies using passive sensing on smartphones, with 18 (51%) studies on mental health: five (14%) on bipolar disorder; five (14%) on depression; and three (9%) on schizophrenia. Other domains included sleep (6; 17%) and general health (4; 11%). Seven studies integrated passive sensing in behavior change interventions such as personalized feedback to promote exercise and healthy eating. Other studies used passive sensing to demonstrate the ability to capture or monitor data related to health and wellbeing. | Bipolar disorder, schizophrenia, MD |
| De Angel 2022 | TRACE DATA | Accelerometer, Light, Electrodermal activity, Skin temperature, Heart Rate, Gyroscope, Accelerometer, Compass, Screen unlock duration, Screen unlock times. Twenty-nine studies collected data on sleep, typically ascer- tained using accelerometer, light and heart rate sensors. Nine different features of sleep are reported. Sleep quality, encompassing features relating to sleep fragmentation (number of awakenings and wake after sleep onset [WASO]), was the most commonly reported feature. Sleep efficiency is presented as a separate feature given its prevalence in studies. For all significant results, lower sleep efficiency or quality was associated with higher depression scores. Sleep Physical activity Circadian rhythm Sociability Location Phone use Physiological Environmental | NI | MDD | MDD |
| De Vries 2020 | EMA/TRACE DATA | 31 studies (58.5%) included objective trace data: accelerometer, GPS location, mobile phone use, heart rate variability, exposimeter of radiofrequency-electromagnetic fields, light, microphone, events, screen use, application use and mobile camera use) and Wi-Fi | 30 studies assessed happiness with a single-item question, 53 studies measured well-being in different contexts (in relation to physical activity and/or sedentary behavior, cognitive processes (e.g. visual search, emotion detection), health, alcohol, mind wandering, food, work, sleep, 2 studies focused on fluctuations of well-being in daily life. Single studies measured momentary well-being in the context of homesickness , phone use, immigrants, soccer, music, fitness exposure, transport and social interactions | The 53 studies measured well-being in different contexts. Momentary well-being in relation to natural environmental variables and contexts (e.g. nature, daylight, urban areas) was assessed in 11 studies. Well-being was assessed in relation to physical activity and/or sedentary behavior (k = 9), cognitive processes (e.g. visual search, emotion detection), health, alcohol, mind wandering, food, work, and sleep. Two studies focused on fluctuations of well-being in daily life . Single studies measured momentary well-being in the context of homesickness, phone use, immigrants, soccer, music, fitness exposure, transport and social interactions. The goal of three studies was to predict momentary mood based on objective data and compared this to EMA data. Finally, one study compared different sampling strategies to assess the effect of context on mood. | Subjective well-being, happiness, quality of life, life satisfaction, positive affect |
| Jameel 2021 | EMA/TRACE DATA | 23 studies used the de- vice for the purpose of assessment and monitoring. Devices were also used to collect passive data from mobile phones (13 studies) or a wearable smartwatch. These devices were used to assess digital social communication, physiology and activity levels. Nine studies used passive methods to assess daily functioning, such as phone call and messaging analysis (e.g. number received/made/sent, length and number of unique numbers), and activity levels (e.g. phone use or GPS data). | 23 studies used the device for the purpose of assessment and monitoring. These studies sought to understand difficulties with functioning and/or to promote self-monitoring. 18 of these used EMAs, self-monitoring software or apps via an mHealth device (mobile smartphone or PDA). ESM studies commonly included people with schizophrenia spectrum conditions and explored the nature of momentary psychotic experiences; whereas self-monitoring studies tended to track changes in mood over time in people with bipolar disorder. As such, self-monitoring studies were typically conducted over much longer periods compared to ESM. 15 papers were classified as intervention studies, which sought to improve an aspect of daily functioning and in some cases symptoms of SMI. Six of the intervention studies used augmented ESM or self-monitoring with feedback to improve functioning or meet daily goals. Three studies used SMS messaging alongside an intervention, to provide individualized prompts and support daily goals. The majority of intervention studies were categorized as blended (i.e. embedded within a broader clinical intervention and/or on-going input from a clinician alongside mHealth device use). Intervention studies included a range of additional blended components, from online psychoeducational modules to individual or group therapy sessions delivered online or in person. | Daily functioning: daily activities and social interactions (i.e., engagement in daily life activities such as working, doing laundry), location (e.g., at home) and social context (e.g., alone), impairment experiences; Feasibility: Fears about using mHealth devices, concerns about data security, confidentiality and storage (e.g. being monitored or identified) Time consuming (i.e. for studies which required regular self-assessment, monitoring or tasks) Other concerns included: Difficulties with accessing the mHealth device and/or software (e.g. compatibility) Preference for face to face vs remote clinical contact (e.g. in intervention studies where the device was used in a standalone manner) Other reasons were also provided which were unrelated to the study or device; Acceptability was assessed via interviews or questionnaires enquiring about people’s experiences of using the mHealth device. The questions enquired about: ease, comfort or difficulties using the mHealth device; burden of using the mHealth device (e.g. disruption to daily activities, time spent completing tasks); embarrassment or other fears about using the mHealth device; how helpful the participants found using it; and their willingness to continue using the device in the future. | Bipolar disorder type I or II, schizophrenia or a schizophrenia spectrum condition, and a mixture of both conditions. |
| Kivela 2022 | EMA | Momentary assess- ments, mood logs, actigraphy data such as daily steps, GPS-derived activity, sleep, heart rate, light sensors recordings, SMS length and count, phone call data, key- stroke meta-data, one’s geocoded activity, and speech technology. Considering the empirical evidence of assessment of depression via digital technology, dimensions of depres- sive symptoms (i.e., mood, psychomotor activity, social functioning, sleep, and cognitive performance) were captured using a variety of digital technologies. These included smartphone-facilitated momentary assess- ments, mood logs, actigraphy data such as daily steps, GPS-derived activity, sleep, heart rate, light sensors recordings, SMS length and count, phone call data, key- stroke meta-data, one’s geocoded activity, and speech technology. | The duration of EMA monitoring ranged from 4 to 60 days (median = 14, n = 23). The number of (scheduled) EMA prompts per day ranged from 1 to 11 (median = 5, n = 21). In adolescent samples, suicidal ideation was reported by 34–82% of the sample during EMA (median = 71%, n = 3), and overall, 2–39% of observations had suicidal ideation ratings >0 (median: 25% n = 3). These thoughts occurred once a week on average, and typically lasted 1 to 30 min [based on a binary measure of ideation]. In adult samples, ideation was reported by 26– 100% of the participants (median = 97%, n = 7), and 1–82% of observations had suicidal ideation ratings > 0 (median: 22% n = 7). While the majority of studies recruited participants with heightened risk profiles (such as those recently discharged after a suicide attempt), prevalence rates in two community-based samples with current self-reported ideation were comparable to the pooled prevalence rates (86–100% participants and 20–22% of all entries indicated suicidal ideation. When examined separately, higher levels of passive (m = 4.54, sd = 2.25, range 2– 10) than active (m = 3.18, sd = 1.50, range 2–10) suicidal ideation was reported. The correlation between depression scores (incl. a suicidal ideation item) derived from the traditionally administered Patient Health Questionnaire-9 [PHQ-9 (93)] and EMA administered PHQ-9 was r = 0.84. EMA-measured momentary suicidal ideation correlated highly with the BSSI [passive ideation: r = 0.73, active ideation: r = 0.76 (67)]. Correlations were higher for items assessing active (“Wish to die” r = 0.76) rather than passive ideation (“Wish to live” r = 0.37). A one-item EMA measure (“How suicidal are you right now?”) correlated highly with the BSSI (r = 0.71) and moderately with the Beck Depression Inventory [BDI] [r = 0.41]. Variability in momentary SI correlated moderately with the Suicide Behaviors Questionnaire - Revised [SBQ-R] (r = 0.41), the BSSI (r = 0.49), and the Capability for Suicide Questionnaire [GCSQ (97)] (r = 0.30). More severe depressive symptoms were reported through EMA than with a traditional retrospective questionnaire, and EMA reports of suicidal ideation were notably higher than questionnaire scores for 69% of the participants. In an adolescent sample, suicidal ideation was reported in EMA by 71% of the participants, and in 45% of the interviews post-EMA. Among adults, 58% of participant reporting SI in EMA did not do so in an interview post-EMA. | More severe depressive symptoms were reported through EMA than with a traditional retrospective questionnaire, and EMA reports of suicidal ideation were notably higher than questionnaire scores for 69% of the participants. In an adolescent sample, suicidal ideation was reported in EMA by 71% of the participants, and in 45% of the interviews post-EMA. Among adults, 58% of participant reporting SI in EMA did not do so in an interview post-EMA. | Suicidal thoughts |
| Kulkarni 2022 | EMA/TRACE DATA | Data collection was conducted using two approaches: actively collected data and passively sensed data. As the name suggests, active data collection involved collecting user input and relied on user compliance. On the other hand, passive data collection used the embedded device sensors to collect user data and relied less heavily on user input and compliance. The majority of the 71 studies (84.5%) used both types of data collection, while the remainder used either active (7%) or passive (8.5%) data. The types of active and passively collected data are discussed in more detail in the following subsections. The majority of studies (93%) utilized passively sensed data, by using smartphone sensors for data collection. While there were a few common in-built hardware sensors such as the accelerometer and GPS/location sensor used in most studies (62% and 53%, respectively), some also looked at software features such as application usage. Studies used a range of sensors and software features to collect various types of contextual information. Besides these conventional types of sensor and software features, several used less common approaches to infer health conditions. For example, two studies used custom key- boards to collect keystroke/keypress data—one to analyze the sentiment of typed text, and the other to infer alcohol intoxication. One study used the barometer sensor for activity recognition and found that it reduced the misclassification of stair-climbing/descending activity. One study examined the correlation between internet-usage data and PHQ (patient health questionnaire) scores, which provide an indication of depression. Fi- nally, one study explored if social media data (Twitter and Instagram) could provide insight into the mental health of individuals. | Studies collected information about a variety of metrics to obtain momentary insights about the users. These included factors such as food intake, perceived loneliness, mood, and stress, to name a few. Although the questions are not standardized across studies (unlike clinical questionnaires), these too are useful in understanding the user contexts. |  | MDD, bipolar disorder, schizophrenia, chronic health conditions. |
| Lo 2020 | TRACE DATA | Type of metrics collected (multiselect): Number of clicks, Number of features used, Number of log-ins, Number of modules, Number of page views, Number of posts, Number of sessions, Rate of return, Session duration, Other (eg, calculated metrics) | ‘Labeling’ passively sensed data and obtaining ground truth which aids in prediction processes using machine learning. | Examination of the efficacy or effectiveness of the intervention in supporting individuals (eg, symptoms) as their primary objective. Studies that included other constructs (eg, feasibility,  acceptability) as primary objectives were typically in the earlier stages of development. These interventions also targeted a range of mental health–related conditions, with depressive disorders as the most common condition (n=13), followed by interventions targeting participants’ overall well-being (n=11). Other targeted conditions included anxiety disorders (n=2), family violence (n=1), feeding and eating disorders (n=6), neurodevelopmental disorders (n=1), schizophrenia spectrum and other psychotic disorders (n=4), substance use and addictive disorders (n=6). | Mental health |
| Marengo 2020 | TRACE DATA | 1) user demographics (typically extracted from Facebook personal information section, including gender, age, education, etc.); 2) activity statistics (e.g. number of posts, number of friends or network density, number of received Likes, comments, and user tags); 3) Likes (e.g. Likes expressed to specific Facebook pages); 4) features derived from the analysis of language in text (e.g. features extracted using closed and/or open-vocabulary approaches); 5) features derived from pictures (e.g. features extracted from uploaded pictures); 6) use of multiple vs. a single type of digital footprints. |  | Big Five personality traits, cognitive processes (e.g. visual search, emotion detection) | Personality |
| Melcher 2020 | EMA/TRACE DATA | GPS, Accelerometer, Call Logs, SMS/email, Screen time, Microphone, Lock/ Unlock events, Light sensors, App Usage, Bluetooth, Browser history | 13 (52%) of studies in this analysis included some active data collection but other types of EMAs such as journals or mobile photographic metres were also used. | Anxiety, stress, loneliness, depression | Mental health |
| Moore 2016 | EMA | NI | Cognitive assessments: Eight studies administered a single mobile cognitive task for the duration of the study, while the other four studies administered more than one task. Tasks were unique in each study; while similar tasks were observed across some studies, no two studies used the same version of a given task. | Cognitive domains assessed included: working memory (n = 7), attention/reaction time (n = 4), processing speed (n = 2), semantic memory (n = 2), short‐term memory (n = 1), delayed memory (n = 1), and executive functions (n = 1). | Cognitive ability |
| Seppala 2019 | TRACE DATA | m-RESIST, sensor -based data in TRS ,The features supplied by sensor data that are used to trigger the CDSS are as follows: app number and duration of incoming, outgoing, and missed calls; number of incoming and outgoing SMS text messages by mobile phone; amount of time spent at home and in other places, measured by GPS data; and amount of time sleeping measured by physiological heart rate | NI | Depression, anxiety, sleep disorders, psychotic disorders, stress, and panic disorders | Schizophrenia, bipolar disorder, and depression |
| Sequeira 2020 | EMA/TRACE DATA | (1) Mobile phones: a passive analytics: including measures such as call logs for incoming and outgoing calls (i.e. call time/date, duration, and contact/ relationship to participant), text message events (i.e. time/date and contact), screen time (i.e. length of on/off, time/date), app use (i.e. name of app launched, when, and for how long), and mobile phone camera use; ( (5) Mobile phones + wearables: a movement and light: including measures such as an individual's current activities and location | 3) physical health: including energy, alcohol use, cannabis use, quality and quantity of sleep, diet, quantity and type of exercise (4) mental health: measuring include affect (I-PANAS-SF), mood,clinical mood domains, stress, recent stressful events, responses to stressful events, formal help seeking, informal help seeking, depression, anxiety, mental state, and coping strategies | Depression in children and/or adolescents | DEPRESSIVE SYMPTOMS |
| Thunnissen 2021 | EMA | NI | Most of the EMA studies included in our review focused on examining the phenomenology of psychopathology and its correlates in daily life, and employed various EMA applications in youths with ADHD, ASD, mood disorders, anxiety disorders, and eating disorders. | EMA was used to provide information about the phenomenology of ADHD by assessing fluctuations, temporal patterns, and contexts of affect and symptomatic behavior, also in the parent– child interactions in daily life. phenomenology of autism spectrum disorder (affect, coping, and related social and behavioral contexts). mood disorders (context of problematic thoughts, A limited number of the included studies used EMA when evaluating treatment outcomes | For example, the NIMH Research Domain Criteria (RDoC) project “integrates many |
| Trifan 2019 | TRACE DATA | accelerometer, GPS, Wi-Fi, Bluetooth, Microphone, gyroscope,calls and text messages, smartphone usage such as screen events, light values, time spent on the phone, and device settings, Google APIs, camera, magnetometer,interaction patterns and near locations, eg bluetooth | NI | 29.6% (35/118) are dedicated to the detection of users’ physical activities. Most of them aimed to recognize basic daily activities such as walking, standing or sitting, jogging or running , going up and down the stairs, lying down, and driving a bike or vehicle. In addition, one study tried to infer riding up and down an elevator, one assessed different activities including being stationary, limping, shuffling, and skipping, and one detected shopping and dining activities. Physical activities were also explored in the sense of detecting and counting steps, distinguishing physical activity from lifestyle activities such as eating, assessing mobility in the elderly to avoid sedentary lives, studying its relationship with happiness including nonexercise activities, or even measuring and predicting the walking speed and distance of patients with pulmonary diseases. sociability was less studied ). Only 5.9% (7/118) of the selected papers chose to infer users’ sleep by detecting sleep patterns, irregular nights, and sleep start and end times. Finally, 10.1% (12/118) of the selected studies developed monitoring systems specifically dedicated to students, mainly to understand how their behaviors (physical activities, sleep, and social interactions) affect their academic performance, mental health, social anxiety, mobility, and behaviors. | Stress conditions, bipolar disorder, anxiety, schizophrenia, depression, psychotic relapse, mood, and affect |
| Verslius 2016 | EMI | NI | EMI - A range of different intervention techniques were studied: CBT, acceptance and commitment therapy, mindfulness, behavioral activation, relaxation, interpersonal therapy, dialectical behavior therapy, cognitive bias modification, and self-management and/or monitoring strategies. The EMI was offered in combination with therapy in 10 studies (30%). | A range of different intervention techniques were studied: CBT acceptance and commitment therapy, mindfulness, behavioral activation, relaxation, interpersonal therapy, dialectical behavior therapy, cognitive bias modification, and self-management and/or monitoring strategies. The EMI was offered in combination with therapy in 10 studies (30%). Four studies combined the EMI with CBT, three with virtual reality including both relaxation and exposure, one with a problem-skill training, one with psychoeducation, and one with meetings including mindfulness and acceptance exercises. In five studies, the EMI was a stand-alone intervention in combination with care as usual. This care focused on bipolar disorder, schizophrenia or schizoaffective disorder, MDD, and alcohol dependency, or on borderline personality disorder and substance abuse. The other 18 studies investigated whether the use of an individual EMI can be effective without face-to-face therapy confounding the effect. Nevertheless, support by an MHP was included in five of these 18 studies. | anxiety disorders, depressive symptoms, stress, bipolar disorder, schizophrenia, borderline personality disorder, bulimia, quality of life |
| Weizenbaum 2020 | EMA/TRACE DATA | Time of day (morning, afternoon, and evening), the quality and amplitude of environmental noise, and the impact of recent social and physical activity on individuals’ task performance. | Semantic reasoning and memory, Working memory, executive function, and languages, Motor speed, attention, and processing speed, reaction time, Processing speed tasks, Memory and executive function, Attention and working memory tasks, Attentional bias | Model of mobile assessment of intraindividual variability in cognition. Internal state-driven variables include affect motivation and alertness. External contextual variables include time of day, social environment, physical surroundings, and physical activity. Taken together, these factors give rise to fluctuations in cognitive performance. This can be captured in real time using a game-like smartphone assessment of cognition alongside sensing tools such as a smartphone microphone and GPS, which seamlessly capture information about one’s environment. | Cognition |
| Zarate 2022 | EMA/TRACE DATA | GPS, accelerometer/actigraphy, Wi-Fi location, screen activity, light exposure, keystroke metadata etc. Digital phenotyping passive sensing, Speech technology and facial recognition. The reviewed studies used a wide variety of methods employing digital means to obtain phenotypical expres- sions of individuals’ depressive symptoms, including self-report apps, smartphone keystroke metadata, mobile phone calls and texts, online surveys, actigrphy sensor-related recordings (i.e., steps, sleep, circa- dian rhythm, GPS), and even digital records of voice. A common rationale to collect data via digital technology involved reflecting on the ubiquitous presence of mobile phones facilitating self-report momentary assessments and highlighting the potential to obtain otherwise elusive information on one’s social engagement behavior | Different aspects of depression focusing on affective, somatic, and cognitive changes in participants: e.g., Mood, cognitive style, social functioning, depression risk and protective factors (intervention) | Depression, e.g., mood, cognitive capacity, suicidal thoughts, sleep–wake cycle, stress, physical activity, depression severity, social interaction, etc.) | Depression |
